# An Injectable Thermosensitive Chitosan/Astaxanthin/Ibuprofen Hydrogel Mitigates High-Voltage, Low-Current Electrical Burn Injury Through Inhibition of ROS–NF-κB Signaling-Mediated Inflammation

**DOI:** 10.3390/pharmaceutics18030323

**Published:** 2026-03-03

**Authors:** Xiao Yang, Hui Wang, Wenjuan Zhang, Peng Gao, Xudong Yu, Weijia Qing, Ping Deng, Jingdian Li, Yan Luo, Li Tian, Jia Xie, Mengyan Chen, Zhengping Yu, Huifeng Pi, Ting Liu, Shenglin Luo

**Affiliations:** 1 Department of Occupational Health (Key Laboratory of Electromagnetic Radiation Protection, Ministry of Education), State Key Laboratory of Trauma and Chemical Poisoning, Army Medical University (Third Military Medical University), Chongqing 400038, China; 17699136712@163.com (X.Y.); zhangxinyue1121@126.com (W.Z.); gaopeng999666@126.com (P.G.); joydeng66@163.com (P.D.); lijingdian_tmmu@163.com (J.L.); rebecca20240815@163.com (Y.L.); 15213201397@163.com (L.T.); 18083020994@163.com (J.X.); 13983292783@163.com (M.C.); yuzping_tmmu@126.com (Z.Y.); pihuifeng@tmmu.edu.cn (H.P.); 2 State Key Laboratory of Trauma and Chemical Poisoning, Institute of Combined Injury, Chongqing Engineering Research Center for Nanomedicine, College of Preventive Medicine, Army Medical University (Third Military Medical University), Chongqing 400038, China; yuxudong2000@126.com; 3 Department of Plastic and Cosmetic Surgery, Xinqiao Hospital, Army Medical University (Third Military Medical University), Chongqing 400038, China; wangh_97@163.com; 4 Dermatology Department, General Hospital of Xizang Military Area Command, Lhasa 850000, China; 5 The 63710th Military Hospital of PLA, Xinzhou 710000, China; gaofeng2018@163.com; 6 Department of Dermatology, Southwest Hospital, Army Medical University (Third Military Medical University), Chongqing 400038, China

**Keywords:** high-voltage and low-current, electrical burns, wound healing, hydrogel, anti-inflammation, reactive oxygen species

## Abstract

**Background/Objectives**: High-voltage, low-current electric shocks inflict superficial second-degree burns on the skin, accompanied by a vicious cycle of excessive oxidative stress and inflammation. As efficient treatment of such electrical burns remains a clinical challenge, we explored the efficacy of an injectable thermosensitive chitosan hydrogel engineered with an antioxidant agent (astaxanthin) and an anti-inflammatory agent (ibuprofen) for the treatment of high-voltage, low-current electrical burn injuries. **Methods**: The proposed CS/AST/IBU hydrogel was prepared and its thermosensitivity was characterized. Subsequently, the hydrogel was injected into the wounds of male Sprague–Dawley (SD) rats subjected to electrical burn injury (20 kV, 3 mA). Finally, a series of experiments were performed to elucidate the dynamics of wound healing and the mechanisms by which the hydrogel promotes wound repair. **Results**: The injectable hydrogel, through its thermally responsive gelation effect at 37 °C, adapts to the complex irregularities of the wound surface. This facilitates the release of astaxanthin and ibuprofen throughout the wound, which collectively diminish the formation of reactive oxygen species and MDA. Furthermore, it enhances the synthesis of endogenous antioxidants such as SOD, CAT, and GSH; encourages collagen deposition; stimulates the development of dermal appendages; and fosters neovascularization. It interrupts the deleterious cycle of oxidative stress and inflammation mediated by the NF-κB signaling pathway, thereby suppressing the expression of pro-inflammatory markers such as TNF-α, CD11b, and IL-1β while upregulating CD163, an anti-inflammatory receptor. **Conclusions**: The use of this multipronged, contour-adaptive hydrogel represents an effective strategy for complex wound management and demonstrates broad therapeutic potential for superficial second-degree electrical burns caused by high-voltage, low-current discharge.

## 1. Introduction

Electrical burns occur when electric currents traverse human skin, engendering thermal effects that lead to injuries such as electrical contact burns, arc burns, and lightning (electrical strike) burns [1]. Electrical burns are characterized by irregular wound morphology, significant hemorrhaging, and the accumulation of free radicals, which collectively contribute to a complex healing microenvironment [2]. Data from the World Health Organization indicate that in developed nations, the annual hospitalization rate attributable to electrical burns constitutes approximately 0.4–5% of overall burn cases; meanwhile, in developing countries, this figure can soar to 27% [3]. In China, the incidence of electrical burns in hospitalized burn patients from 1 January 2013 to 31 December 2022 was around 10% [4]. More specifically, high-voltage, high-current electrical burns often lead to extensive distal necrosis of the limbs, including third-degree burns with damage to muscles and loss of digits [5]. Unlike high-voltage, high-current electrical burns, those caused by close-range contact with uninsulated equipment (e.g., electric batons or other non-lethal electric shock weapons) are primarily limited to the superficial layers of both the epidermis and dermis [6]. To date, advancements in the treatment of electrical burns have been made, particularly in fluid resuscitation, complication prevention, and wound healing. However, the multitude of complications (e.g., infection, scar formation, and shock) prolong hospitalization and impose substantial burdens on patients and healthcare systems [7]. Although expedient wound closure is a crucial component in the treatment of electrical burns [8], this process is frequently hindered by aberrant inflammatory responses that derail the normal healing cascade, with dysregulation of the local immune microenvironment impairing re-epithelialization and slowing tissue regeneration [9,10]. Therefore, developing advanced biomaterials capable of modulating these inflammatory pathways is a critical therapeutic strategy.

Clinically, the superficial second-degree burns caused by high-voltage, low-current electric shocks do not mandate aggressive surgical intervention; however, the absence of appropriate management exacerbates pain and increases the risk of injury propagation [11]. Accordingly, there is a compelling demand for minimally invasive, non-surgical modalities for this purpose, ideally possessing self-adaptive properties to accommodate the irregular wound topographies inherent to electrical injuries. Self-adaptive therapeutic biomaterials are increasingly being engineered to facilitate minimally invasive and personalized wound management. Injectable or moldable hydrogel formulations ensure conformal coverage of complex, deep, or irregular wound topographies, thereby obviating the necessity for adjunctive surgical preparation. Upon exposure to physiological stimuli—including temperature, ionic strength, or shear force—these systems undergo in situ gelation via sol–gel transition or shear-thinning mechanisms to immediately establish a protective matrix [12]. This versatility enhances clinical precision across a broad spectrum of pathologies, ranging from burn injuries to infected traumatic wounds. Therefore, these conformable systems promote individualized therapeutic regimens and extend the applicability of advanced wound care to refractory injuries.

Thermoresponsive biomaterials, which are driven by intermolecular forces, have recently emerged: these materials undergo a reversible sol–gel phase transition at a critical temperature, leading to a volumetric change and transformation into a semi-solid hydrogel [13,14]. Specifically, dynamic crosslinking mediated by hydrophobic interactions and hydrogen bonds allows the material to undergo phase separation in solution below the critical temperature and rapid gelation above the critical temperature [15]. Chitosan (CS), with its high porosity, biodegradability, and predictable degradation kinetics, serves as an excellent scaffolding backbone for smart polymers [16,17]. Hydroxypropyl methylcellulose (HPMC) can be crosslinked with CS to not only to enhance the structural stability of CS but also to provide a reversible, temperature-dependent sol–gel transition [18,19]. Notably, the inclusion of glycerol tunes the lower critical solution temperature (LCST) of HPMC from approximately 60 °C to physiological temperature (37 °C), ensuring rapid in situ gelation upon contact with the wound [20]. This property allows the formulation, which is liquid at ambient temperature, to be injected to fill intricate electrical burn defects. It subsequently becomes a semi-solid gel that establishes intimate contact with the wound bed for sustained drug delivery [21]. Furthermore, the inherent radical scavenging properties and resistance to oxidative degradation of chitosan contribute to its broad utility in biomedical applications, including in oncology and wound healing [22,23].

In this work, we engineered a thermoresponsive hydrogel by co-loading astaxanthin (AST) and ibuprofen (IBU) into a chitosan framework. AST, a xanthophyll carotenoid predominantly found in aquatic fauna, functions as a potent natural antioxidant for cytoprotection against oxidative damage [24]. Notably, compared with β-carotene and lutein, AST is more effective at inhibiting UV-induced lipid photooxidation, with an antioxidant capacity greater than that of α-tocopherol (vitamin E) and β-carotene [25,26]. Ibuprofen (IBU), a canonical non-steroidal anti-inflammatory drug, is poorly water-soluble, and its therapeutic efficacy is contingent upon sustained, low-dose release [27]. Thus, we incorporated these antioxidant and anti-inflammatory drugs into an injectable, thermoresponsive hydrogel, designated CS/AST/IBU. When evaluated in a skin electrical burn model, this hydrogel significantly accelerated wound closure and attenuated the local inflammatory cascade. Mechanistically, proteomics and Western blotting revealed that the therapeutic efficacy of the CS/AST/IBU hydrogel was driven by the inhibition of excessive peroxide levels and the potent downregulation of NF-κB signaling. In conclusion, the dual ability of the CS/AST/IBU thermosensitive hydrogel to both physically adapt to high-voltage, low-current electrical burn defects and biochemically regulate the wound microenvironment positions it as a comprehensive strategy for promoting advanced wound healing.

## 2. Materials and Methods

### 2.1. Materials

All the chemicals were purchased from Aladdin (Shanghai, China) and used as received.

### 2.2. Preparation of CS/HPMC Hydrogel Precursor Solution

Glacial acetic acid was diluted with deionized water to create a 0.5% (*v*/*v*) aqueous solution of acetic acid. For preparation of the hydrogel matrix, chitosan (CS; 100 mg; degree of deacetylation: 85%) and hydroxypropyl methylcellulose (HPMC; 700 mg; viscosity: 6 mPa·s) were weighed and blended thoroughly to produce a uniform powder. A volume of 10 mL of the 0.5% acetic acid solution was placed in a water bath and heated to 80 °C, following which the CS/HPMC powder was added. The resulting mixture was vortexed for 1 min and subsequently subjected to ultrasonic treatment for 5 min. The mixture was continuously stirred on a magnetic stirrer at 80 °C for 30 min, until it had fully dissolved and reached optical clarity. After cooling to room temperature, the pH of the mixture was adjusted to approximately 6.8 with a 2 mol L^−1^ sodium hydroxide solution. Multiple batches of the CS/HPMC hydrogel precursor solution were prepared and stored at 4 °C until required.

### 2.3. Preparation of AST/HPCD Complex

To prepare the inclusion complex, astaxanthin (AST; 10 mg; ≥98% HPLC) was first dissolved in 10 mL of dichloromethane (DCM). This organic phase was then introduced into a methanolic solution (40 mL) of hydroxypropyl-β-cyclodextrin (HPCD) at a concentration of 25 mg/mL. The system was sealed under a nitrogen atmosphere, sonicated for 5 min to ensure homogeneous dispersion, and subsequently agitated at 35 °C for 48 h in the dark. Following this incubation, the solvents were removed via vacuum concentration. The resulting solid residue was redissolved in deionized water and purified via vacuum filtration to remove insoluble, uncomplexed AST. The final orange-colored filtrate was collected, flash-frozen, and subsequently lyophilized to yield a fine powder of the AST/HPCD complex (986 mg).

### 2.4. Preparation of IBU/β-CD Complex

The inclusion complex was synthesized using a solvent evaporation method. Ibuprofen (100 mg) and β-cyclodextrin (β-CD; 748 mg) were co-dissolved in 30 mL of ethanol at a 1:1 stoichiometric molar ratio. The mixture was heated at 90 °C in a water bath for 60 min to ensure complete dissolution, then cooled to 40 °C and subjected to continuous magnetic stirring for an additional 3 h. The solvent was subsequently removed under reduced pressure (i.e., via rotary evaporation) to yield a solid white residue. For purification, this residue was redissolved in deionized water and subjected to vacuum filtration to eliminate any uncomplexed, water-insoluble ibuprofen. The final clear filtrate containing the soluble complex was flash-frozen and lyophilized to obtain the purified product as a fine, white IBU/β-CD powder (844 mg).

### 2.5. Preparation of CS/AST/IBU Hydrogel

The CS/HPMC hydrogel precursor solution (10 mL) was meticulously added to glycerol (4.7 mL) and gently stirred until a uniform solution emerged. This mixture was designated as the CS hydrogel. Separately, the CS/HPMC hydrogel precursor solution (10 mL) was combined with 100 mg of the IBU/β-CD complex under gentle stirring, followed by the gradual addition of glycerol (4.7 mL), which was then termed the CS/IBU hydrogel. In another instance, the CS/HPMC hydrogel precursor solution (10 mL) was blended with 100 mg of the AST/HPCD complex under gentle stirring, followed by the dropwise incorporation of glycerol (4.7 mL) until homogeneity was reached, and thus labeled as the CS/AST hydrogel. Finally, the CS/HPMC hydrogel precursor solution (10 mL) was concurrently mixed with 10 mg of the IBU/β-CD complex and 90 mg of the AST/HPCD complex, for a total inclusion complex content of 100 mg, followed by dropwise addition of glycerol (4.7 mL) at a volume fraction of 32% until a homogeneous solution was attained (with a final total volume of 14.7 mL). This resultant mixture was identified as the CS/AST/IBU hydrogel.

Subsequently, in vitro drug release studies were performed using a dialysis bag method. Briefly, 1 mL of the CS/AST/IBU hydrogel was placed into a dialysis membrane (MWCO 3500 Da) and immersed in 20 mL of PBS (pH 7.4) containing 0.5% Tween-80 to maintain sink conditions. Release experiments were conducted at 25 °C and 37 °C with constant shaking (100 rpm). At predetermined time intervals, 1 mL of release medium was withdrawn and replaced with fresh medium. The concentrations of IBU and AST were determined using UV–Vis spectrophotometry based on standard curves. The cumulative release percentage was calculated and plotted against time. Release data were fitted to zero-order, first-order, Higuchi, and Korsmeyer–Peppas models to elucidate the release kinetics and mechanisms.

The drug encapsulation efficiency (EE) was calculated using the following equation: Encapsulation Efficiency (EE, %) = W_encapsulated_/W_feed_ × 100%.

W_encapsulated_: The actual mass of pure drug in the lyophilized cyclodextrin inclusion complex after vacuum filtration to remove any unencapsulated free drug that was not bound to cyclodextrins.

W_feed_: The initial mass of pure drug added for preparation of the inclusion complex (i.e., 100 mg IBU, or 10 mg AST respectively).

### 2.6. Characterization of the CS/AST/IBU Thermosensitive Hydrogel

The CS/AST/IBU thermosensitive hydrogel was quenched via immersion in liquid nitrogen for 3 min, after which the quenched samples were freeze-dried for 24 h for scanning electron microscopy (SEM) analysis. The gel sections were sprayed with gold and characterized via SEM (JSM-5900LV model, JEOL, Tokyo, Japan) at an electron accelerating voltage of 20 kV.

### 2.7. Determination of the Lower Critical Solution Temperature (LCST)

The LCST was determined using the inverted tube method. The prepared solution was transferred into glass test tubes and placed in a thermostatically controlled water bath at a specified temperature. After 10 min, the tubes were removed and inverted to observe fluid mobility. The temperature at which no flow occurred was recorded as the minimum gelation temperature. If flow persisted, the temperature was incrementally increased by 1 °C, with observations made every 10 min until the LCST was identified.

### 2.8. Fourier Transform Infrared (FTIR) Spectroscopy

The samples were dried at 100 °C, ground to a fine powder, and mixed with potassium bromide (KBr) to prepare pellets. FTIR spectra were acquired using a Spectrum GX spectrometer (PerkinElmer, Shelton, CT, USA) over the range 4000–500 cm^−1^ with a resolution of 4 cm^−1^.

### 2.9. Rheological Analysis

Rheological measurements of the CS/HPMC/glycerol system were performed using a DHR-1 rheometer (TA Instruments, New Castle, DE, USA) with a 40 mm parallel-plate geometry and a plate gap of 1 mm. A measured volume of the sample was placed between the plates, and the temperature was increased from 20 to 80 °C to determine the relationship between the viscoelastic modulus and temperature. Subsequently, frequency sweep tests were conducted at a constant temperature of 37 °C to determine the gelation kinetics and calculate the gelation time under physiologically relevant conditions.

### 2.10. Cell Culture and In Vitro Biocompatibility Assay

HaCaT cells (cas No. iCell-h066) were acquired from ICell Bioscience Inc. (Shanghai, China). The cells were maintained in high-glucose Dulbecco’s Modified Eagle Medium (DMEM; Corning, Corning, NY, USA; 10-013-CVRC) enriched with 10% fetal bovine serum (FBS; Ausgenex, Molendinar, Australia; FBS500-s) and 1% penicillin/streptomycin (GIBCO, Waltham, MA, USA; 15140-122). HaCaT cells in the logarithmic growth phase were harvested and seeded into 96-well plates at a density of 8 × 10^3^ cells per well. The cultures were maintained in a humidified incubator at 37 °C with 5% CO_2_. Subsequently, the cells were subjected to treatments according to predefined experimental groups: the control group received 100 μL/well of complete medium, while the Sample group was treated with 100 μL/well of working solutions at varying concentrations (200 μL of the hydrogel sample was immersed in 4 mL of culture medium for extraction for 24 h). All experimental conditions were performed in triplicate. Following 24 h of incubation at 37 °C with 5% CO_2_, the culture medium was aspirated and the wells were rinsed 3 times with phosphate-buffered saline (PBS). A 100 μL aliquot of fresh medium supplemented with 10% Cell Counting Kit-8 (CCK-8) reagent was then added to each well. After a 2 h incubation period, the absorbance was quantified at 450 nm using a microplate reader.

### 2.11. Animals and Treatments

A total of 60 male Sprague–Dawley (SD) rats (6 weeks of age, SPF grade) were obtained from the Experimental Animal Center of Army Medical University. The rats were healthy, free of genetic modifications, and had no prior experimental treatment. The animals were housed in ventilated cages under controlled environmental conditions (temperature: 22 ± 2 °C; relative humidity: 50–60%) with a housing density of three rats per cage. Following a 1-week acclimatization period, all rats were randomly allocated to fice experimental groups (12 rats per group) using a random number table. This sample size was determined to satisfy the requirements of statistical tests, based on the effect size (d = 1.5) obtained from the pilot experiment and the sample size calculation performed using G*Power 3.1 software (α = 0.05, β = 0.2). The calculation results indicated that three animals per group were required for each time point.

The inclusion criteria were 6-week-old SPF grade SD rats weighing 180–220 g; rats with abnormal body weight or lethargy were excluded. During the experiment, cages were randomly placed, and all experimental treatments were uniformly performed between 8:00 and 12:00 a.m. daily, with measurements conducted in the order of control groups followed by experimental groups. All rats in each group remained in good condition and were included in the final analysis.

The experimental design specified that three rats from each group would be sacrificed and sampled at 0, 1, 3, and 5 days after the electrical injury. The specific groupings were as follows: the negative control group (control group) comprised SD rats subjected to electrical injury with no subsequent treatment; the experimental control group (CS group) included SD rats with electrical injury covered solely with pure hydrogel; and the three experimental groups consisted of SD rats with electrical injury treated with hydrogel mixed with astaxanthin (CS/AST group), hydrogel mixed with ibuprofen (CS/IBU group), and hydrogel co-loaded with both astaxanthin and ibuprofen (CS/AST/IBU group). Following observation in the preliminary experimental model, the control group and the CS/AST/IBU group (six rats per group, obtained from the Experimental Animal Center of Army Medical University) were selected for re-modeling experiments involving proteomic and Western blot analyses to elucidate the underlying molecular mechanisms.

The experimental design, procedures, and euthanasia methods were reviewed and approved by the Institutional Animal Welfare and Ethics Committee of Army Medical University (AMUWEC20226182, Approval date: 31 October 2022). Strict adherence to blinding protocols was maintained throughout the study, with both the experimenters performing the procedures and the outcome assessors evaluating the results remaining blind to group allocations. The data analyst was also kept blind until the statistical analysis was fully completed, at which point unblinding was carried out in accordance with standardized protocols. To ensure the objectivity and reliability of the results, group allocation confirmation and data graphing were independently conducted by two researchers after the study data were locked and finalized, with cross-validation performed to eliminate potential biases.

### 2.12. Animal Anesthesia

In the present study, a fractional intraperitoneal injection protocol was adopted for experimental rats. In particular, an initial dose of 30 mg/kg was administered [28], followed by close observation of changes in corneal reflex and muscle tone in the rats. When the rats failed to reach the required anesthetic depth for electrical injury induction, an additional dose of 10 mg/kg was supplemented, resulting in a total anesthetic dose of 40 mg/kg [29,30]. After treatment, the rats were transferred to a warm recovery cage and observed until complete awakening (ability to move freely and take food/water). Postoperative care was performed for 24 h, including body temperature monitoring, diet management, and wound inspection to ensure animal welfare and experimental reliability.

### 2.13. Establishment of Skin Electrical Burn Injury and Hydrogel Intervention

Following hair removal from the gluteal region and right hind limb, the animals were secured in the supine position. One electrode was positioned on the skin overlying the ventral belly of the gastrocnemius muscle, 0.5 cm adjacent to the right ankle malleolus, at a vertical distance of 0.5 mm from the skin surface. A second electrode was placed on the skin over the ipsilateral gluteal muscle at an identical vertical distance (0.5 mm) from the skin surface. According to the preset experimental groups, controlled electrical stimulation was delivered to the right hindlimb. In this study, an electric baton (K98, Jiangsu Colin, Suzhou, China; 20 kV, 3 mA) was employed to induce electrical burns. Energy deposition was predominantly concentrated within the epidermis and superficial dermis, resulting in moderate tissue damage mediated by electrothermal and electrostimulation effects, distinct from the penetrating thermal injury characteristic of high-voltage trauma. Following stimulation, irregular lesions with surface areas ranging from 2.36 to 2.62 mm^2^ were observed. The electrical discharge, characterized by high voltage and low amperage (20 kV, 3 mA), was applied for a duration of 5 s, consistent with our previously established protocol [31].

The wound sites of the rats were first disinfected with iodophor. After the wounds were air-dried, a 30 G needle was used to slowly inject the hydrogel at a low-dose of 2 μL per site into the superficial wound bed (dermal papillary layer) through the wound edge (instead of the central lesion). The needle was inserted obliquely at a 45° angle to avoid deep puncture injury to viable dermal appendages, and gentle pressure was applied to the injection site after injection to prevent cavity formation and mechanical overload on adjacent healthy tissues.

Rats were euthanized via carbon dioxide (CO_2_) inhalation, which complies with animal ethics requirements and international standards for laboratory animal welfare. Subsequently, the rats were quickly fixed in a prone position and the skin on both thighs was thoroughly disinfected with 75% medical ethanol. The skin was incised along the lateral midline of the thigh, and the subcutaneous tissue was separated from the muscle layer. Skin tissue samples (approximately 1 cm × 1 cm in size) were harvested from the injured area and the surrounding 1 cm region. After harvesting, surface blood and contaminants of the tissue were immediately rinsed with normal saline, and the attached fat and fascia tissues were removed. Tissue samples were promptly excised and either fixed in 4% paraformaldehyde (PFA) or stored at −80 °C in an ultra-low temperature freezer for subsequent experimental assays.

### 2.14. Antioxidant Performance of the Hydrogel

The antioxidant capacity of the hydrogel was evaluated by investigating its ability to scavenge the 1,1-diphenyl-2-picryl-hydrazyl radical (DPPH), 2,2′-azinobis-(3-ethylbenzthiazoline-6-sulfonate) (ABTS), superoxide anion radicals (•O_2_^−^), and hydroxyl radicals (•OH), according to the manufacturer’s instructions.

### 2.15. Histological Examination and Immunofluorescence Staining

Wound surfaces were observed and documented on days 0, 1, 3, and 5 after model induction. In accordance with the established literature [32,33], accurate depth assessment in experimental burn models is contingent upon stabilization of the tissue injury, which effectively occurs subsequent to a 24 h post-injury latency. Given that diverse diagnostic modalities demonstrate improved efficacy in differentiating burn severities beyond this temporal threshold, skin tissue samples were harvested at 24 h post-injury to definitively characterize burn depth. The ratio of wound area on day 5 to that on day 1 was employed as the primary injury evaluation index to quantify wound healing efficacy. Meanwhile, wound tissues were harvested, rinsed thoroughly with phosphate-buffered saline (PBS), and fixed in 4% paraformaldehyde solution for subsequent experiments. The tissues were embedded in paraffin and sectioned into 5 μm thick slices for histological and immunohistochemical analyses, including hematoxylin and eosin (H&E) staining, Elastica van Gieson (EVG) staining, Masson’s trichrome staining, immunofluorescence (IF) staining, and immunohistochemistry (IHC). H&E, Masson’s trichrome, and IF staining procedures were performed according to the manufacturers’ standardized protocols. The tissue sections were deparaffinized in xylene and rehydrated through a graded ethanol series. For immunofluorescence staining, the sections were incubated with primary antibodies overnight at 4 °C, followed by a 30 min incubation with secondary antibodies at 37 °C. For detailed information on the antibodies, please refer to Table S1.

### 2.16. Proteomic and Bioinformatic Analysis

Proteins were extracted from tissue samples using SDT lysis buffer (4% SDS; 100 mM DTT; 100 mM Tris-HCl, pH 8.0). The samples were subsequently boiled for 3 min, followed by ultrasonic disruption. Insoluble cellular debris was removed via centrifugation at 16,000× *g* for 15 min, and the supernatant was collected and quantified using a BCA protein assay kit (BeyoTime, Haimen, China). Briefly, detergents, DTT, and IAA were added to UA buffer to block reduced cysteines. Finally, the protein suspensions were digested overnight at 37 °C with trypsin (Promega, Madison, WI, USA) at a 50:1 enzyme-to-protein ratio. Peptide mixtures were collected via centrifugation at 16,000× *g* for 15 min, desalted using C18 StageTips, and subjected to LC-MS analysis. The concentration of reconstituted peptides was determined by measuring the OD280 using a Nanodrop One device (Thermo, Waltham, MA, USA).

DIA MS data were analyzed with DIA-nn 1.8.1, and MS spectra were searched against the UniProtKB/Swiss-Prot database with trypsin specified as the digestion enzyme. Database searches allowed for a maximum of one missed cleavage site and a mass tolerance of 10 ppm for both precursor and fragment ions. Cysteine carbamidomethylation was defined as a fixed modification, whereas protein N-terminal acetylation and methionine oxidation were variable modifications (a maximum of 1 variable modification per peptide). Peptides were restricted to lengths of 7–30 amino acids and charge states of 1–4, with fragment ions detected within an m/z range of 150–2000. The database search results were filtered with a false discovery rate (FDR) of <1% at both the peptide-spectrum match and protein levels before export.

Bioinformatic analyses were performed using Microsoft Excel and R-4.4.1 statistical software. Hierarchical clustering analysis was performed, and volcano plots were generated using R. Sequences were annotated with information from the UniProtKB/Swiss-Prot, the Kyoto Encyclopedia of Genes and Genomes (KEGG), and Gene Ontology (GO) databases. GO and KEGG enrichment analyses were conducted using Fisher’s exact test with FDR correction for multiple comparisons. GO terms were categorized into biological processes (BPs), molecular functions (MFs), and cellular components (CCs). Enriched GO terms and KEGG pathways were considered nominally significant at a *p*-value < 0.01 according to Fisher’s exact test.

### 2.17. Western Blot Analysis

Skin tissue samples were harvested and homogenized in radioimmunoprecipitation assay (RIPA) buffer supplemented with protease inhibitors to obtain total protein lysates. The protein concentration of each lysate was quantified using a bicinchoninic acid (BCA) protein assay kit according to the manufacturer’s instructions. Equal amounts of protein (e.g., 20–30 µg) from each sample were resolved via electrophoresis on 12% SDS-polyacrylamide gels (SDS-PAGEs) and subsequently transferred to polyvinylidene difluoride (PVDF) membranes. The membranes were blocked for 1 h at room temperature with 5% non-fat milk or bovine serum albumin (BSA) in Tris-buffered saline containing 0.1% Tween-20 (TBST) to minimize non-specific antibody binding. Following blocking, the membranes were incubated with the NF-κB P65 antibody (cas No. AB2020, Beyotime; 1:1000) overnight at 4 °C. After washing with TBST, the membranes were incubated with the appropriate horseradish peroxidase (HRP)-conjugated secondary antibodies (anti-rabbit or anti-mouse) for 1 h at room temperature. The protein bands were visualized using an enhanced chemiluminescence (ECL) detection system. GAPDH (cas No.AF1186, Beyotime, 1:1000) was used as an internal loading control for normalization.

### 2.18. Statistical Analysis

Statistical analyses and graphical representations were performed using the GraphPad Prism 8.0 software. All quantitative data are presented as the mean ± standard error of the mean (SEM), derived from three independent experimental replicates. The normality of data distributions was verified via the Shapiro–Wilk test prior to parametric analysis. Subsequently, intergroup differences were evaluated using two-tailed unpaired *t*-tests for dual comparisons or one-way analysis of variance (ANOVA) followed by Tukey’s post hoc test for multiple group comparisons. A *p*-value of < 0.05 was considered statistically significant.

## 3. Results

### 3.1. Synthesis and Physicochemical Characterization of the CS/AST/IBU Hydrogel

A thermoresponsive matrix was first formulated through physical crosslinking of the CS backbone with HPMC and glycerol, driven by non-covalent interactions. This biocompatible matrix was subsequently loaded with astaxanthin (AST) and β-cyclodextrin-encapsulated ibuprofen (IBU) to engineer a therapeutic hydrogel which is capable of scavenging reactive oxygen species (ROS) and exerting anti-inflammatory effects, thereby promoting accelerated wound repair.

**Figure 1:**
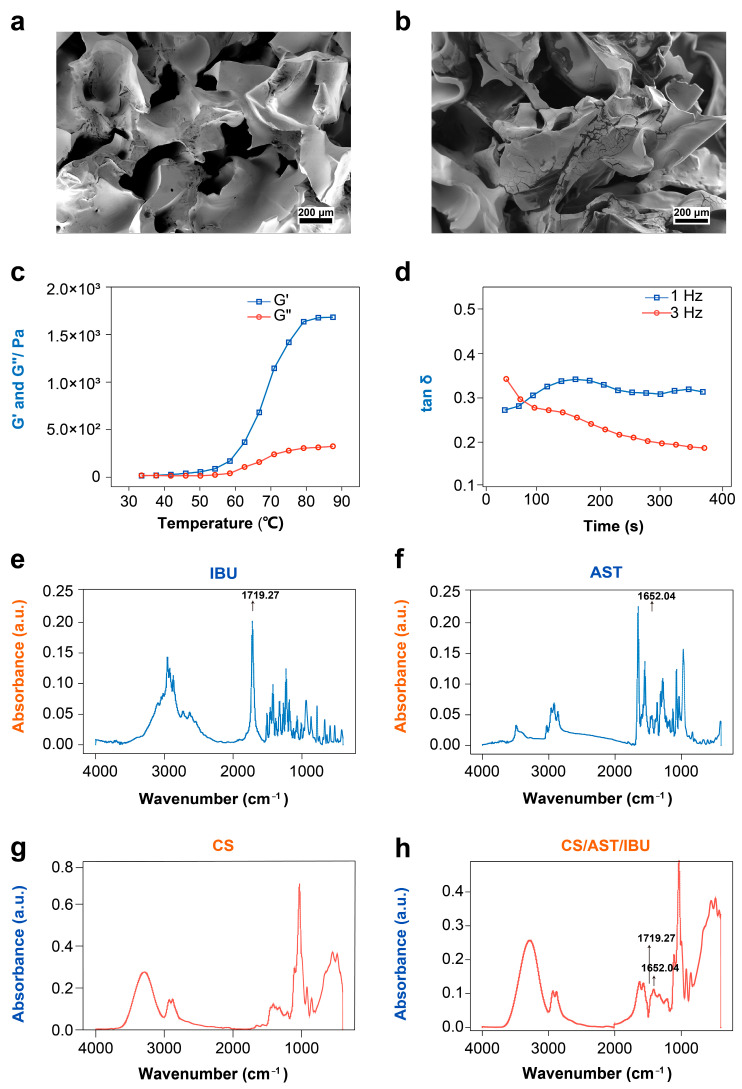
Physicochemical characteristics of the CS/AST/IBU hydrogel. SEM images of (**a**) CS hydrogel and (**b**) the CS/AST/IBU hydrogel. (**c**) Rheological properties of the CS/AST/IBU hydrogel across various temperatures. (**d**) Gelation duration of the specimen at 37 °C. The absorption rates of infrared light by (**e**) IBU, (**f**) AST, (**g**) CS, and (**h**) CS/AST/IBU hydrogel.

For effective wound dressings, a microstructure that supports cellular infiltration and adhesion is paramount. Scanning electron microscopy (SEM) of the hydrogel revealed a highly interconnected, three-dimensional porous architecture (Figure 1a,b). As illustrated in Figure 1c, during the initial heating phase, both the storage modulus (G′) and the loss modulus (G″) of the solution were relatively low, indicating good fluidity, expressed as G′ = G″. When the temperature increased above 30 °C, G′ increased sharply whereas G″ decreased slightly, suggesting a transition in the fluid dynamics of the solution. At 37 °C, G′ and G″ diverged, with G′ surpassing G″ and maintaining G′ > G″ thereafter, thereby indicating a sol–gel transition within the system at this temperature. The gelation time of the sample at 37 °C is depicted in Figure 1d. The tanδ (G″/G′) values stabilized at varying frequencies, signifying that tanδ was no longer influenced by frequency fluctuations. The intersection of the tanδ values indicates complete gel formation. Over time, tanδ increased at 1 Hz, whereas it decreased at 3 Hz. At approximately 1500 s, the rate of change in tanδ abruptly slowed, indicating pre-gelation of the sample (Phase 1). The intersection point at approximately 800 s suggests that the CS/AST/IBU hydrogel oil solution had fully gelled at this time (Phase 2)—a duration significantly longer than the visually observed gelation time.

To elucidate the underlying molecular interactions, we performed Fourier transform infrared (FTIR) spectroscopy (Figure 1e–h). The spectrum of pure CS showed the expected broad –OH and –NH stretching bands (3100–3400 cm^−1^) and characteristic amide I and III bands (1657 and 1319 cm^−1^, respectively), where the low intensity of the amide bands aligns with the specified high degree of deacetylation (85%). In the composite materials, the narrowing of the hydroxyl band and attenuation of peaks in the 1200–1500 cm^−1^ region indicate intermolecular hydrogen bond formation among CS, HPMC, and glycerol, confirming that the network is held together by physical interactions rather than covalent crosslinking. Crucially, the successful incorporation of the therapeutic payloads was confirmed by the appearance of characteristic vibrational signatures in the final spectrum of the CS/AST/IBU hydrogel. A prominent peak at 1719.27 cm^−1^ is attributed to the C=O stretching vibration of the carboxylic acid in IBU, whereas a distinct signal at 1652.04 cm^−1^ corresponds to the conjugated C=C system of AST, verifying the entrapment of both agents within the polymer matrix.

### 3.2. In Vitro Drug Loading and Release

**Figure 2:**
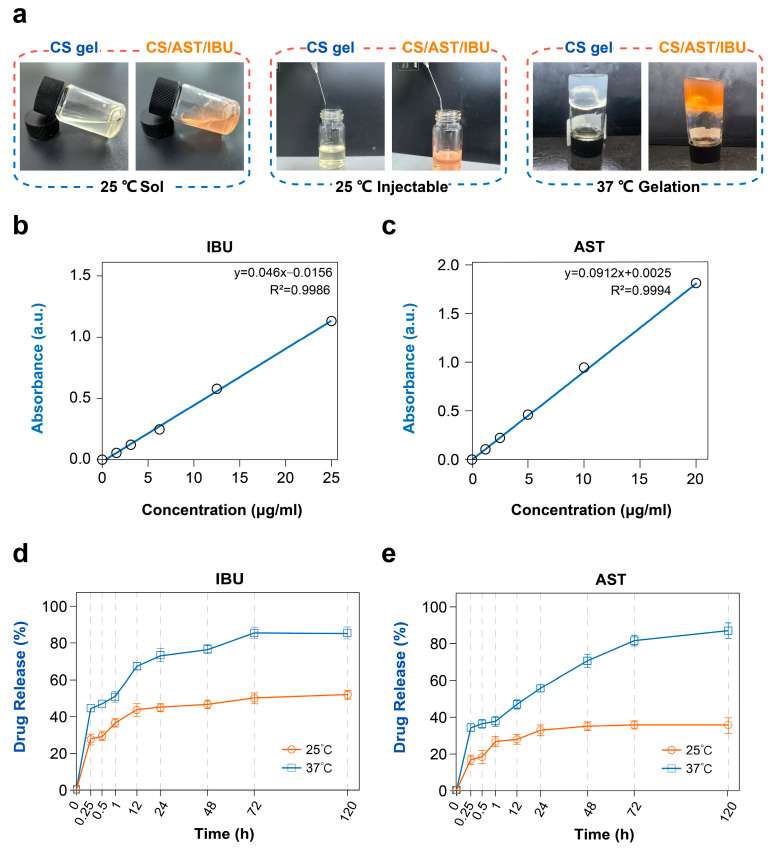
Thermosensitive characteristics of the CS/AST/IBU hydrogel. (**a**) Morphology of the CS gel and CS/AST/IBU hydrogel at varying temperatures. Concentrations of (**b**) IBU and (**c**) AST in the CS/AST/IBU hydrogel. Release rates of (**d**) IBU and (**e**) AST from the CS/AST/IBU hydrogel at various temperatures.

The hydrogel was designed to exhibit an obvious, physiologically relevant sol–gel transition. Notably, at ambient temperature (25 °C), the CS/AST/IBU hydrogel exhibits a distinct liquid and injectable consistency (Figure 2a). Upon heating to physiological temperature (37 °C), the hydrogel undergoes gelation and transitions to a gelatinous state. According to the established standard curves of IBU and AST (Figure 2b,c), increasing concentrations of AST or IBU correlate with higher absorbance values, indicating a linear relationship that enables the quantification of drug concentrations via fitting of the calibration curve. The drug content of ibuprofen in the ibuprofen β-cyclodextrin inclusion complex was 52.56 ± 1.0 μg/mg, derived from the mass of encapsulated IBU relative to the total mass of the lyophilized inclusion complex (844 mg), with an encapsulation efficiency (EE) of 44.11 ± 0.84%. The drug content of Astaxanthin in the Astaxanthin β-cyclodextrin inclusion complex was 4.14 ± 0.3 μg/mg, derived from the mass of encapsulated AST relative to the total mass of the lyophilized inclusion complex (986 mg), with an encapsulation efficiency (EE) of 40.82 ± 2.96%. Therefore, for the total drug content in the whole formulation, the 14.7 mL injectable hydrogel contained a total of 525.6 ± 10 μg of IBU (10 mg × 52.56 ± 1.0 μg/mg) and 372.6 ± 27 μg of AST (90 mg × 4.14 ± 0.3 μg/mg). For the drug content per unit volume, consistent with the drug loading concentration above, the formulation contained 35.55 ± 0.68 μg IBU and 25.35 ± 1.84 μg AST per mL. Regarding the actual drug dosage per single injection, the hydrogel was injected at a low-dose of 2 μL per site into the superficial wound bed (dermal papillary layer) through the wound edge using a 30 G needle in the animal experiment. Thus, the actual drug dosage per single injection at each site was 0.0711 ± 0.00136 μg of IBU (35.55 ± 0.68 μg/mL × 2 μL) and 0.0507 ± 0.00368 μg of AST (25.35 ± 1.84 μg/mL × 2 μL). Comparative analysis of drug release profiles at 25 °C and 37 °C revealed that the AST and IBU release efficiency increased as the temperature exceeded the lower critical solution temperature (LCST). Elevated temperatures disrupted hydrogen bonding between CS molecules and water, enhancing hydrophobic interactions within CS chains. Above 37 °C, these hydrophobic forces coupled with residual hydrogen bonding drove the formation of a stable hydrogel network. To gain deeper insight into the release mechanisms, the in vitro release data of IBU and AST at 37 °C were fitted to various kinetic models, as summarized in Supplementary Table S2. Based on the correlation coefficients, the drug release data of the hydrogel at 37 °C exhibited a better correlation with the Korsmeyer–Peppas model [34]. These n values fall within the range of 0.45–0.89, indicating that drug release from the CS/AST/IBU hydrogel follows an anomalous (non-Fickian) transport mechanism. This is consistent with the thermosensitive nature of the hydrogel; at 37 °C, the polymer matrix undergoes gelation, and drug release is governed by a combination of Fickian diffusion and polymer chain relaxation. It is important to note that while the release mechanism is non-Fickian, the overall release rate is relatively rapid. Approximately 70% of the IBU and 55% of the AST were released within the first 24 h at 37 °C. We believe that this release profile is biologically rational for the treatment of acute electrical burns. The initial burst facilitates rapid establishment of therapeutic concentrations of both the anti-inflammatory agent (IBU) and the antioxidant (AST) during the critical early phase of wound healing (first 1–3 days), when oxidative stress and inflammation are most severe. The subsequent slower release phase helps to maintain these effects and supports the later stages of tissue repair. This in vitro release profile correlates well with the in vivo efficacy observed in our animal model, where significant improvements in re-epithelialization and neovascularization were evident by day 5. Collectively, the CS/AST/IBU hydrogel achieved a reasonable rate of drug release (Figure 2d,e).

### 3.3. Antioxidant Performance of the CS/AST/IBU Hydrogel

**Figure 3:**
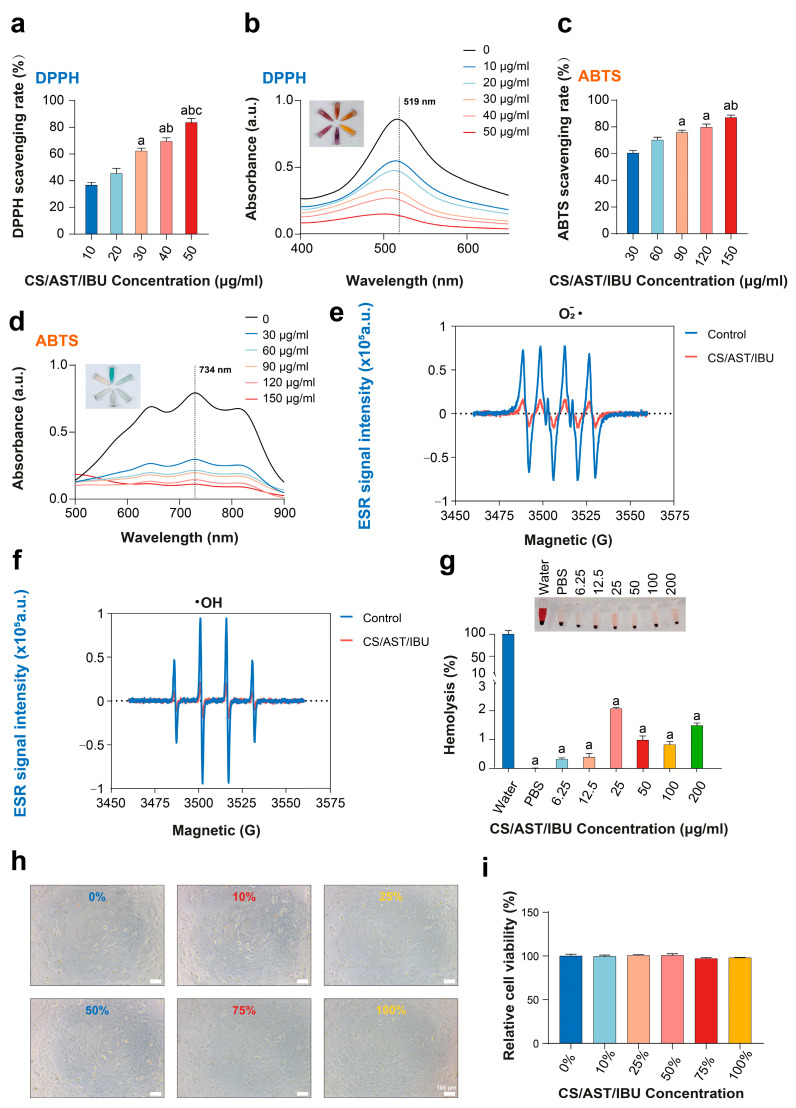
Antioxidant performance, biocompatibility, and cytotoxic effects of the CS/AST/IBU hydrogel. (**a**–**d**) Scavenging efficiency of the CS/AST/IBU hydrogel towards two typical free radicals (DPPH and ABTS), monitored using UV-Vis spectroscopy and quantitatively analyzed. (**a**) For statistic difference, in (a) a: *p* < 0.05 versus 10 μg/mL, b: *p* < 0.05 versus 20 μg/mL, and c: *p* < 0.05 versus 30 μg/mL; in (**c**) a: *p* < 0.05 versus 30 μg/mL and b: *p* < 0.05 versus 60 μg/mL. ESR spectra of (**e**) O_2_•^−^ and (**f**) •OH. (**g**) Hemolysis rate of the CS/AST/IBU hydrogel; a: *p* < 0.05 versus water. (**h**) Microscopy images of HaCaT cells cultured with the CS/AST/IBU hydrogel. (**i**) Results of the CCK-8 assay indicating HaCaT cell viability after culture with the CS/AST/IBU hydrogel. (Shapiro–Wilk and Brown–Forsythe tests for normality and variance homogeneity, respectively. One-way ANOVA with Tukey’s post hoc analysis for multiple groups).

Chronic wounds resulting from electrical burns frequently exhibit excessive accumulation of reactive oxygen species (ROS), which impair cellular proliferation and tissue remodeling, thereby delaying wound repair [35]. Biomaterials endowed with ROS-scavenging capacity can alleviate oxidative stress and facilitate tissue regeneration. In this study, we systematically evaluated the efficiency of the CS/AST/IBU hydrogel in scavenging DPPH, ABTS, •OH, and O_2_•^−^ radicals. As the CS/AST/IBU gel concentration increased from 10 to 50 μg mL^−1^, DPPH scavenging increased from 38% to 82%, accompanied by strong absorbance at 519 nm (Figure 3a,b). Similarly, increasing the concentration from 30 to 150 μg mL^−1^ increased ABTS radical scavenging from 60% to 85%, with a pronounced increase in absorbance observed at 734 nm (Figure 3c,d).

Free radicals, which are chemical species containing unpaired electrons, generate characteristic signals in electron spin resonance (ESR) spectroscopy. As such, ESR spectroscopy is widely employed to study radicals and vacancies, considering that these species are intricately linked to chemical reactions and material properties. As illustrated in Figure 3e,f, electron paramagnetic resonance (EPR) analysis demonstrated the potent free radical scavenging capacity of the CS/AST/IBU hydrogel. Compared with the control, the hydrogel induced a significant reduction in the peak-to-peak amplitude of the EPR signals, with a 78.0% reduction for the superoxide radical (•O_2_^−^) and a 78.3% reduction for the hydroxyl radical (•OH). This indicates its effective ability to neutralize both types of reactive oxygen species. Detailed kinetic parameters are provided in Supplementary Table S3. For the DPPH assay, CS/AST/IBU solutions were prepared at five concentrations (10, 20, 30, 40, and 50 μg/mL), whereas the ABTS assays utilized concentrations of 30, 60, 90, 120, and 150 μg/mL; 0 μg/mL served as the negative control in both experiments. The results showed that CS/AST/IBU exhibited concentration-dependent ROS-scavenging activity, with maximal efficacy observed at 50 μg/mL (DPPH) and 150 μg/mL (ABTS).

### 3.4. Biocompatibility of the CS/AST/IBU Hydrogel

Prior to further application, the in vivo cytotoxicity of the CS/AST/IBU gel was evaluated through assessment of hemolysis in mouse peripheral blood. At concentrations ranging from 6.25 to 200 μg/mL, the gel exhibited hemolysis rates below the critical 5% threshold, indicating favorable hemocompatibility for use as a blood-contacting therapeutic material [36] (Figure 3g). As the cytocompatibility of the CS/AST/IBU hydrogel is a critical prerequisite for its clinical translation, we assessed its potential in vitro cytotoxicity by coculturing the material with human immortalized epidermal cells (HaCaT cells). Quantitation using a cell counting kit-8 (CCK-8) assay demonstrated that the hydrogel possessed robust cytocompatibility, as cell viability was maintained above 95% across all tested concentrations, indicating no discernible cytotoxic effects (Figure 3h,i).

This finding is particularly important given that high concentrations of ibuprofen have been reported to induce cytotoxicity through activation of the mitochondrial apoptotic pathway [37]. In our engineered formulation, no such adverse effects were observed. We attribute this favorable safety profile to two fundamental design principles: the intrinsically non-toxic nature of astaxanthin and the effective sequestration of ibuprofen within the physically crosslinked CS/HPMC/glycerol matrix. This encapsulation strategy mitigates the risks posed by direct cellular exposure to high localized concentrations of the anti-inflammatory drug.

### 3.5. The CS/AST/IBU Hydrogel Accelerates Cutaneous Wound Healing In Vivo

The 24 h interval represents a rational and reliable time point for depth assessment in this model, corresponding to stabilization of the tissue injury [32,33]. Histopathological evaluation performed 24 h post-injury revealed structural compromise confined to the epidermal and superficial dermal layers. The tissue manifested characteristic pathological changes, including cellular congestion, edema, and leukocyte infiltration, accompanied by swelling and disordered arrangement of collagen fibers (Supplementary Figure S1). These findings are consistent with the diagnostic criteria for superficial partial-thickness burns resulting from high-voltage, low-current electric shock exposure.

**Figure 4:**
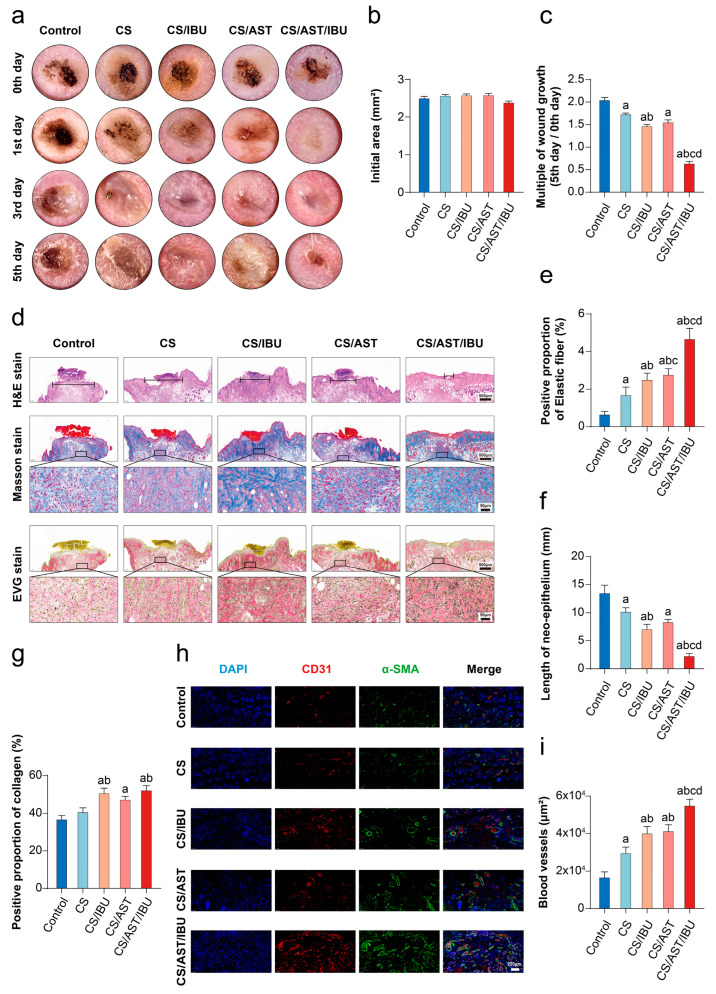
The CS/AST/IBU hydrogel accelerates cutaneous wound healing in vivo. (**a**) Representative images of the wound healing process at various time points in different treatment groups. Quantitative evaluation of the wound surface area after (**b**) 0 and (**c**) 5 days of treatment with CS/AST/IBU hydrogel. (**d**) Optical representations alongside their corresponding amplified depictions of H&E, Masson’s trichrome, and EVG staining of wounds subjected to various treatments. The black lines in H&E staining images represent the length of the neo-epithelium at day 5. In the Masson’s trichrome and EVG staining images, the areas enclosed by the black boxes are magnified and shown below. (**e**) Positive proportion of elastic fibers, (**f**) length of the neo-epithelium, and (**g**) positive proportion of collagen in the various treatment groups. (**h**) Corresponding dual staining of α-SMA and CD31 at day 5 (red: CD31; green: α-SMA) (**i**) Immunofluorescence staining for CD31 and α-SMA of tissue at the wound site was performed on day 5 after treatment to evaluate angiogenesis within the wound tissue. a: *p* < 0.05 versus control (the electrical injury group); b: *p* < 0.05 versus CS; c: *p* < 0.05 versus CS/IBU; and d: *p* < 0.05 versus CS/AST. (Shapiro–Wilk and Brown–Forsythe tests for normality and variance homogeneity, respectively. One-way ANOVA with Tukey’s post hoc analysis for multiple groups).

To evaluate the in vivo therapeutic efficacy of the CS/AST/IBU hydrogel, we established a skin electrical burn rat model (20 kV, 3 mA, 5 s). Healing was observed and documented photographically at 0, 1, 3, and 5 days (Figure 4a). On days 0–1 post-injury, the cutaneous lesions were characterized by epidermal detachment, edema, and coagulative necrosis. By days 3–5, the tissue edema gradually subsided and the inflammatory response was mitigated, marked by the initiation of fibrous connective tissue proliferation. Macroscopic assessment revealed that closure kinetics of the wounds treated with the CS/AST/IBU hydrogel were markedly accelerated. By day 5 post-injury, the residual wound area in the CS/AST/IBU group was significantly smaller than that in both the untreated control and the blank CS hydrogel groups, underscoring the potent pro-regenerative capacity of the multifunctional hydrogel (Figure 4b,c). Furthermore, the tissues from the CS/AST/IBU-treated group displayed superior regeneration, characterized by more complete re-epithelialization and diminished wound gaps. These findings provide compelling evidence that the proposed dual-action hydrogel, engineered for both reactive oxygen species (ROS) scavenging and anti-inflammatory activity, effectively modulates the wound microenvironment to promote constructive tissue repair (Figure 4d,f).

To further investigate the quality of the neotissue, we performed Masson’s trichrome staining to assess collagen deposition and the regeneration of dermal appendages. By day 5, although control wounds still presented with residual eschar, those treated with the CS/AST/IBU hydrogel displayed a well-organized and continuous epidermal layer (Figure 4e,g). Histopathological analysis via hematoxylin and eosin (H&E) staining corroborated these macroscopic findings. Quantitative analysis of the histological sections revealed a significantly higher density of nascent collagen fibers and regenerating skin appendages in the CS/AST/IBU group, indicating a superior quality of tissue remodeling (Figure 4d,e). These data collectively demonstrate that the hydrogel not only accelerates wound closure but also promotes high-quality regenerative healing.

### 3.6. The CS/AST/IBU Hydrogel Promotes Angiogenesis and Vascular Maturation

The formation of a robust vascular network is a hallmark of successful wound healing and is essential for perfusing nascent tissue and supporting the regeneration of dermal appendages. We therefore sought to determine whether the accelerated repair observed in CS/AST/IBU-treated wounds was underpinned by increased angiogenesis. Therefore, we performed immunofluorescence staining of wound biopsies to evaluate neovascularization at the protein level (Figure 4h).

Staining was performed for CD31 and α-SMA, serving as specific markers for endothelial cells and pericytes/smooth muscle cells, respectively. In mature blood vessels, the endothelial cell lumen is enveloped by α-SMA-positive cells. This co-localization pattern is a key morphological hallmark of vascular maturation, stability, and regulative capacity, indicating that the structure evolves beyond a simple endothelial tube into a fully functional vascular unit. Wounds treated with the CS/AST/IBU hydrogel exhibited a significantly higher density of CD31-positive microvessels and a more mature, stabilized vascular plexus than those in the control groups, as evidenced by increased α-SMA expression (Figure 4i). These data strongly suggest that the CS/AST/IBU hydrogel orchestrates an accelerated healing response in burn injuries by creating a pro-regenerative niche—a process driven by the synergistic effects of inflammation suppression and potent stimulation of functional angiogenesis.

### 3.7. Immunomodulatory Action of the CS/AST/IBU Hydrogel

**Figure 5:**
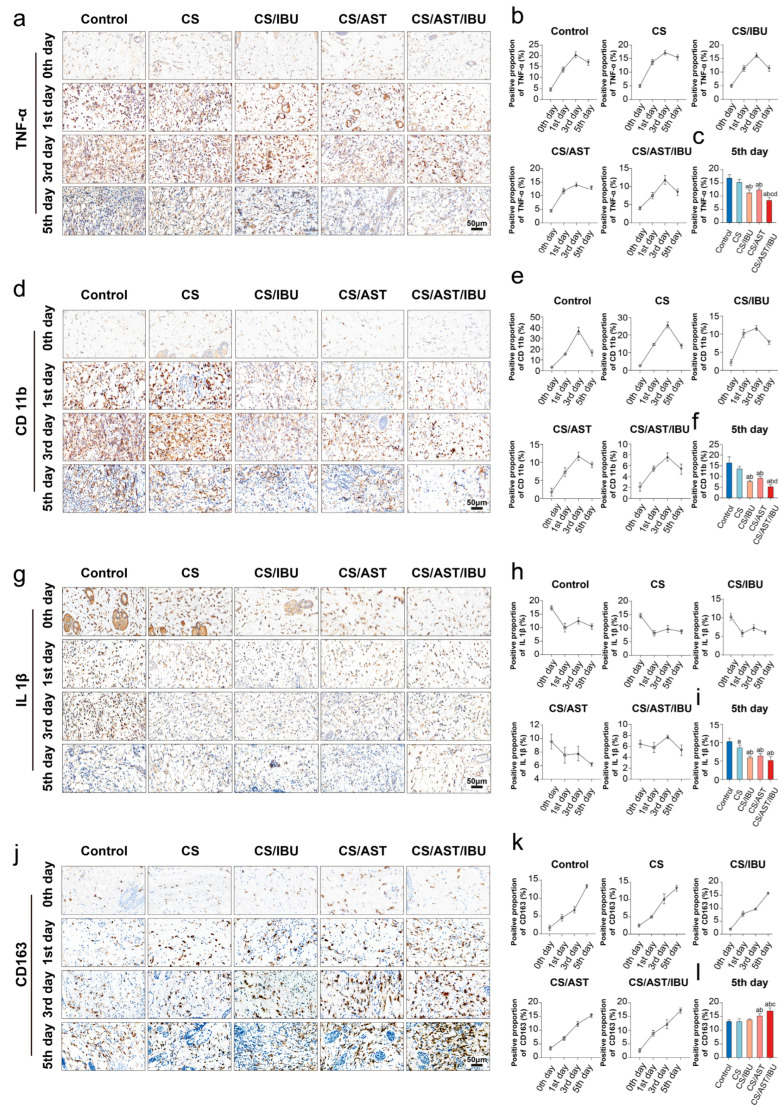
Anti-inflammatory properties of the CS/AST/IBU hydrogel. Immunohistochemistry and quantitative analysis of (**a**–**c**) TNF-α, (**d**–**f**) CD11b, (**g**–**i**) IL-1β, and (**j**–**l**) CD163 expression following hydrogel treatment over various intervals. Brown: target protein; Blue: hematoxylin-stained cell nucleus. Data shows the proportion of protein-positive area per unit stained area under different treatments. a: *p* < 0.05 versus control (the electrical injury group); b: *p* < 0.05 versus CS; c: *p* < 0.05 versus CS/IBU; and d: *p* < 0.05 versus CS/AST. (Shapiro–Wilk and Brown–Forsythe tests for normality and variance homogeneity, respectively. One-way ANOVA with Tukey’s post hoc analysis for multiple groups).

Given that dysregulated and prolonged inflammation is a critical barrier to efficient wound healing, we hypothesized that the observed therapeutic benefit was mediated by the potent anti-inflammatory function of the hydrogel. To investigate the underlying mechanism, we performed immunohistochemical (IHC) staining of key inflammatory markers on wound tissue. The results revealed profound downregulation of the expression of the pro-inflammatory markers TNF-α (Figure 5a–c), CD11b (Figure 5d–f), and IL-1β (Figure 5g–i). Interestingly, we found that CD163—which can indirectly contribute to the anti-inflammatory response—was significantly upregulated (Figure 5j–l) in tissues treated with the CS/AST/IBU hydrogel compared with other groups. This potent suppression of the inflammatory cascade provides a direct mechanistic link to accelerated wound repair, confirming that effective immunomodulation is a cornerstone of the therapeutic efficacy of the hydrogel.

### 3.8. Proteomic Profiling Reveals the Pro-Regenerative Mechanisms of the CS/AST/IBU Hydrogel

**Figure 6:**
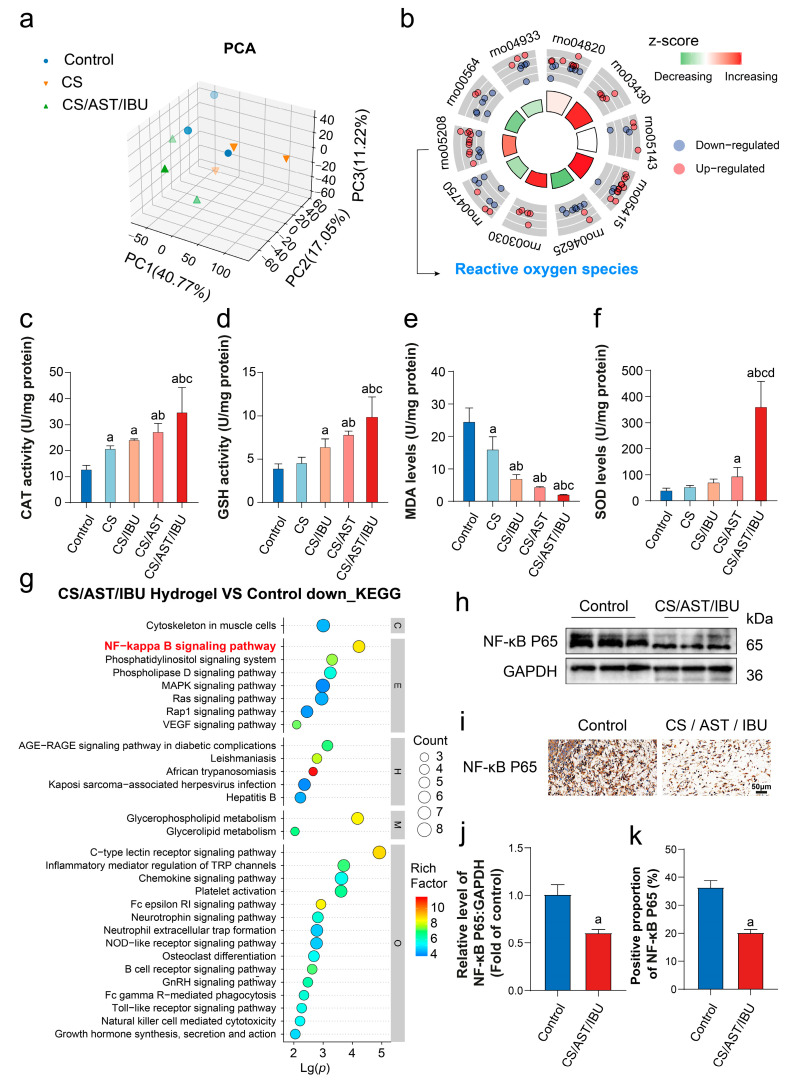
Anti-inflammatory and antioxidant mechanisms of the CS/AST/IBU hydrogel. (**a**) Principal component (PCA) analysis. (**b**) Circle plot depicting the DEPs. Activities of (**c**) CAT and (**d**) GSH and the levels of (**e**) MDA and (**f**) SOD in the different treatment groups. (**g**) KEGG enrichment analysis of the DEPs. Western blot (**h**,**j**) and IHC (**i**,**k**) analyses of the levels of NF-κB-p65. a: *p* < 0.05 versus control (the electrical injury group); b: *p* < 0.05 versus CS; c: *p* < 0.05 versus CS/IBU; and d: *p* < 0.05 versus CS/AST. (Shapiro–Wilk and Brown–Forsythe tests for normality and variance homogeneity, respectively. Unpaired *t*-tests for two groups. One-way ANOVA with Tukey’s post hoc analysis for multiple groups).

To elucidate the molecular mechanisms underlying the therapeutic efficacy of the CS/AST/IBU hydrogel for electrical burn wounds, we performed a label-free quantitative proteomic analysis on wound bed tissues at the study endpoint. The hierarchical clustering heatmap revealed distinct metabolic profiles in injured skin tissues treated with the CS/AST/IBU hydrogel or CS alone, compared with normal tissues (Supplementary Figures S2 and S3). Principal component analysis (PCA) of differentially abundant metabolites confirmed the clear separation of metabolic signatures across the three groups using metabolomic profiling (Figure 6a). Applying thresholds of |log_2_(fold change)| > 1.2 and *p* < 0.05, we identified 405 differentially abundant metabolites between the CS/AST/IBU and control groups, of which 262 were upregulated and 143 were downregulated (Supplementary Figure S4). These differentially expressed metabolites were predominantly categorized into amino acids, nucleotides and their derivatives, and organic acids and their derivatives.

To functionally annotate these DEPs, we conducted Gene Ontology (GO) enrichment analysis and classified the proteins into three primary domains: cellular components (CCs), molecular functions (MFs), and biological processes (BPs). CC analysis revealed that the DEPs were predominantly localized to the intracellular region, whereas the MF analysis highlighted their associations with protein binding, cell adhesion molecule binding, and acyltransferase activity. Critically, the BP analysis demonstrated significant enrichment of proteins integral to constructive biological processes, including cellular component organization or biogenesis, cellular localization, cellular component organization, and cellular component biogenesis (Supplementary Figure S5).

The circle plots prepared from the KEGG enrichment analysis results show the top 10 pathways most significantly associated with the differentially expressed proteins (Figure 6b). Among these enriched pathways, the reactive oxygen species signaling pathway (rno05208) was identified, with 10 upregulated and 4 downregulated proteins. The Z score—calculated as the difference between the number of upregulated and downregulated genes associated with a given GO term divided by the square root of the total number of genes annotated to that term—revealed an increasing trend for the ROS signaling pathway. To elucidate the ROS-scavenging capability of CS/AST/IBU, we quantified the levels of catalase (CAT), glutathione (GSH), malondialdehyde (MDA), and superoxide dismutase (SOD), which are established biomarkers of oxidative stress. In particular, CAT is a critical antioxidant enzyme that decomposes hydrogen peroxide into water and oxygen [38]; GSH is a potent non-enzymatic antioxidant that neutralizes free radicals and protects cells against oxidative damage [39]; MDA, a major product of lipid peroxidation, serves as a marker of oxidative stress-induced membrane injury [40]; and SOD catalyzes the dismutation of superoxide anions (O_2_•^−^) into hydrogen peroxide (H_2_O_2_), reflecting the ability to clear peroxides [41]. Figure 6c–f show that, compared with both the control and CS-only groups, the CS/AST/IBU treatment markedly increased CAT and SOD activities, as well as GSH levels, while significantly reducing MDA content. These findings indicate that the CS/AST/IBU hydrogel effectively scavenges excessive amounts of reactive oxygen species within the wound microenvironment, consistent with our previous ESR results demonstrating substantial elimination of •OH and O_2_•^−^ radicals.

Furthermore, KEGG enrichment analysis revealed that the markedly downregulated proteins were predominantly clustered in pathways such as NF-κB, inflammatory mediator regulation of TRP channels, chemokine signaling, neurotrophin signaling, B-cell receptor signaling, and natural killer cell-mediated cytotoxicity (Figure 6g). These findings suggest that treatment with CS/AST/IBU attenuates signaling cascades associated with inflammation and immune responses, particularly the NF-κB pathway. Collectively, the results of this proteomic analysis provide a comprehensive molecular blueprint for the mechanism of the hydrogel, demonstrating a decisive transition from a state characterized by persistent inflammation and excessive ROS to a microenvironment conducive to active, multifaceted tissue regeneration.

### 3.9. Molecular Validation of the Antioxidant and Anti-Inflammatory Mechanisms

To dissect the molecular underpinnings of the accelerated healing cascade, we employed Western blot and immunohistochemistry (IHC) assays to validate the expression of key regulatory proteins in the signaling pathways identified through our proteomic analysis. Given that excessive apoptosis and a hyperinflammatory state are well-established impediments to wound resolution, our validation focused on central nodes involved in these processes.

Our analysis confirmed decisive modulation of key pathways at the protein level. In wound tissues treated with the CS/AST/IBU hydrogel, we detected a profound reduction in the expression of NF-κB-p65, the canonical transcription factor of the NF-κB inflammatory signaling pathway, according to Western blot (Figure 6h,j; Supplementary Figure S6) and IHC assays (Figure 6i,k).

These results provide a compelling mechanistic rationale for the therapeutic effects of the hydrogel. The concurrent downregulation of ROS, which govern lipid peroxidation, indicates an enhanced anti-inflammatory microenvironment. Critically, the downregulation of NF-κB-p65 expression indicates strong hydrogel-induced attenuation of NF-κB-driven inflammation, mainly due to ROS scavenging. Taken together, these data establish that the CS/AST/IBU hydrogel orchestrates an accelerated healing response by decisively inhibiting peroxidation and resolving inflammation, thereby creating a microenvironment which is conducive to tissue regeneration.

## 4. Discussion

At present, electric burns represent a significant contributor to occupational burn injuries globally [3]. Nevertheless, the medical management of high-voltage, low-current electrical burns—particularly regarding wound healing—continues to pose considerable obstacles. Injuries induced by high-voltage, low-current electric shock exposure are predominantly localized to the superficial epidermal and dermal layers, manifesting as partial-thickness burns in our studies. Although surgical intervention is not needed in such cases, meticulous wound management and dressing coverage remain imperative to address the significant pain and serous exudation associated with these lesions. Hydrogels have drawn intense attention as a research focus in wound repair and are particularly valued for their ability to maintain a moist, slightly hypoxic microenvironment. They can also absorb wound exudates and block microbial invasion, thus preventing secondary injury. In addition, hydrogels offer biodegradability and biocompatibility properties analogous to those of the extracellular matrix [42,43].

Injectable hydrogels present a superior therapeutic modality in burn management compared with conventional preformed dressings [44]. Unlike matrices requiring surgical implantation, injectable hydrogels are delivered via minimally invasive injection to establish a stable three-dimensional crosslinked network mimicking the extracellular matrix (ECM) [45]. This architecture facilitates seamless adaptation to deep and irregular wound geometries, thereby eliminating dead space and circumventing the mechanical compression associated with rigid materials. Thus, local microcirculatory obstruction and secondary ischemic necrosis can be avoided. In our study, in vivo assessments revealed that the CS/AST/IBU hydrogel can significantly augment vascularization processes. CD31-positive microvessel density and α-SMA expression were markedly elevated compared with the control group, suggesting the formation of a mature and stable vascular plexus after treatment. These findings demonstrate that the hydrogel architecture facilitates neovascularization, eliminating the need for mechanical compression that may cause ischemic necrosis. In addition, electrical injury is characterized not only by thermal energy deposition but also by direct electroporation and electrochemical disruption of cell membranes, leading to progressive microvascular thrombosis, compartment syndrome, and delayed tissue necrosis—a phenomenon well-documented in the literature, even in seemingly superficial cases [46,47]. The injection route is designed to bypass the epidermal coagulative necrosis caused by high-voltage, low-current electrical burn injury and ensure conformal coverage of complex, deep, or irregular wound topography [48]. Collectively considering its precise morphological conformity and superior biocompatibility application, this injectable hydrogel represents a potent treatment strategy for superficial burns resulting from high-voltage, low-current electrical exposure.

Inspired by thermosensitive hydrogels, we developed an injectable CS/HPMC/glycerol formulation to address these issues. The CS/HPMC hydrogel has marked advantages; for example, when injected at ambient temperature into elongated wound tracts, the physiological body heat induces rapid gelation, enabling conformal contact with the wound bed while permitting sustained, controlled release of therapeutic agents [14]. Structural analysis confirmed the formation of a stable, highly porous network, serving as an optimal polymeric scaffold for cell adhesion and tissue regeneration. Importantly, when incorporated, the native molecular integrity of both IBU and AST was preserved, with no structural degradation. Spectroscopic characterization revealed the attenuation or disappearance of peaks in the 2500–3000 cm^−1^ range, indicating intermolecular interactions between CS, IBU, and AST, whereas the principal absorption peaks for IBU (1719.27 nm) and AST (1652.04 nm) remained intact, confirming the absence of covalent chemical reactions and the predominance of physical interactions.

Rheological evaluation revealed gelation at 37 °C, while drug release profiling indicated that as the ambient temperature approached the LCST, the release rates increased to a maximal level, with sustained delivery over 6–7 days. These findings collectively verify the successful synthesis of a polysaccharide-based thermosensitive hydrogel system with robust structural integrity, precise LCST tuning to physiological temperature, and compatibility with active agents without compromising their molecular structure. In this study, the viability of HaCaT cells exceeded 95% after treatment with concentrations ranging from 300 to 3000 μg mL^−1^, and the hemolysis rate of the hydrogel remained below the critical threshold of 5% within the range of 6.25–200 μg mL^−1^. The results of the cytotoxicity and hemolytic analyses collectively confirmed the low in vivo toxicity of the CS/AST/IBU composite. Although previous reports have indicated the potential hepatic and renal toxicity of IBU at supratherapeutic doses [49,50], the high drug encapsulation efficiency and slow release rate of the hydrogel effectively minimize its cytotoxicity. Given the close association between biocompatibility and biosafety, these results substantiate that the CS/AST/IBU hydrogel possesses favorable biological compatibility and safety, suggesting its considerable translational potential for clinical application.

Oxidative stress is a pivotal driver of multiorgan dysfunction in the early stages of severe burns [51,52]. In the acute phase, substantial fluid loss and excessive ROS generation can synergize with inflammatory mediators and vasoactive substances to induce profound structural and functional alterations in cells, culminating in severe oxidative injury [53,54]. Furthermore, lipid peroxidation is also strongly associated with the incidence of multiorgan dysfunction and other complications following severe burns [55]. Lipids, which are highly susceptible to ROS attack, can yield lipid radicals (RO•, ROOH) and peroxidation products such as MDA and 4-hydroxynonenal, which further react with cell membranes, proteins, and DNA, thus impairing cellular functions [56]. The incorporation of potent antioxidants capable of donating electrons or stabilizing radical intermediates into hydrogel matrices is therefore of particular importance for post-electrical-burn wound repair.

In this study, the CS/AST/IBU system maintained >60% efficiency in scavenging both DPPH and ABTS radicals within the concentration range of 30–150 μg mL^−1^, corroborating the hypothesis that AST and CS derivatives act cooperatively to eliminate ROS. Mechanistically, AST possesses an extensive conjugated π-electron system, while chemically modified CS contains hydroxyl and phenolic groups, both of which confer robust radical scavenging activity [57,58]. Previous studies have shown that the conjugated polyene backbone of AST, together with its terminal unsaturated keto and hydroxyl groups, exhibits high electron mobility, enabling efficient electron donation or acceptance to neutralize free radicals—an activity markedly exceeding that of common antioxidants [59] and consistent with the results of our present study. Additionally, phenolic hydroxyl modifications to CS has been shown to markedly increase its antioxidant potential [60]. Recent studies have reported that CS derivatives outperform native CS in scavenging DPPH, hydroxyl, and superoxide radicals, primarily through the hydrogen-donating properties of phenolic hydroxyl groups and the cooperative effects of amino and hydroxyl groups along the CS backbone [61]. Finally, IBU exhibits negligible scavenging activity against DPPH or ABTS radicals [62]. Although the antioxidant properties of astaxanthin are well established [63], it should be noted that a positive antioxidant control (e.g., Vitamin C) was not included in the current antioxidant evaluation system. The addition of Vitamin C as a positive control will be considered and supplemented in our subsequent related research to further verify and compare the antioxidant performance of the proposed hydrogel system.

Notably, treatment with the CS/AST/IBU hydrogel, particularly at higher doses, markedly attenuated lipid peroxidation. Improvements included elevated CAT, GSH, and SOD activities, along with reduced MDA levels. The antioxidant effect was more pronounced in the CS/AST group than in the CS/IBU group, underscoring the superior ability of AST to reverse lipid oxidative damage. While CS derivatives alone did not significantly increase GSH or SOD activities, the levels of both proteins tended to increase slightly, suggesting that the inhibitory effect of CS derivatives on lipid peroxidation may not rely primarily on an increase in antioxidant enzyme activity.

Hydrogel dressings have demonstrated substantial promise in tissue regeneration. Nonetheless, achieving optimal therapeutic efficacy in the harsh microenvironment of electrically induced skin injuries remains challenging. Excessive ROS in the electrical burn microenvironment can directly modify cellular constituents or activate transcription factors, thereby perturbing angiogenesis and anti-inflammatory signaling [64]. These mechanisms are consistent with our observations, which indicate that the CS hydrogel system loaded with IBU and AST effectively scavenges ROS and protects wounds from oxidative stress, thereby promoting healing, immune modulation, and angiogenesis. Both in vitro and in vivo experiments revealed that the combined incorporation of IBU and AST markedly accelerated the repair of electrically burned skin tissue.

To elucidate the mechanism underlying this effect, proteomic analysis was performed. KEGG enrichment analysis revealed a significant decrease in the activity of the NF-κB signaling pathway in the CS/AST/IBU group compared with the control group. Western blotting and IHC were subsequently conducted to confirm the results of the KEGG enrichment analysis, and the CS/AST/IBU hydrogel was found to markedly reduce NF-κB expression. Interestingly, many studies have confirmed that astaxanthin inhibits NF-κB activation through multiple mechanisms, including direct suppression of IκBα degradation and blockade of nuclear translocation of the NF-κB p65 subunit [65]. Separately, Li et al. reported that chitosan markedly suppressed NF-κB and TNF-α expression in a rat wound model [66]; however, Cherng J et al. observed a significant increase in NF-κB levels in wound tissue following the application of a chitosan dressing [67]. This apparent discrepancy may be attributable to differences in the molecular weight, degree of deacetylation, or derivative type of chitosan, as well as in the wound-healing stage. In addition, direct evidence confirming modulation of the NF-κB pathway by IBU remains limited. Collectively, these findings suggest that astaxanthin may play a pivotal role in the immunomodulatory properties of the CS/AST/IBU hydrogel.

Moreover, astaxanthin-mediated suppression of NF-κB expression significantly downregulates the production of diverse pro-inflammatory mediators, such as TNF-α, IL-6, IL-8, and inducible nitric oxide synthase (iNOS), ultimately mitigating inflammatory responses [68,69]. By virtue of its central transcriptional regulatory role, NF-κB directly increases the expression of pivotal pro-inflammatory mediators such as TNF-α and IL-1β and indirectly modulates the activities of integrins, including CD11b, through the upregulation of adhesion molecules [70]. Newly synthesized TNF-α and IL-1β further activate NF-κB, establishing a self-amplifying positive feedback loop that drives and sustains a pronounced inflammatory response within the wound microenvironment [71]. In our study, the CS/AST/IBU hydrogel concomitantly decreased TNF-α, CD11b, and IL-1β levels while increasing CD163 expression. These findings indicate that the CS/AST/IBU hydrogel inhibited the ROS–NF-κB–inflammatory axis, resulting in a pronounced shift toward the activation of anti-inflammatory signaling and attenuation of pro-inflammatory signaling within the wound milieu.

Although the CS/AST/IBU hydrogel exhibited potent therapeutic efficacy in repairing second-degree thermal skin burns induced by high-voltage, low-current electrical exposure, there are some limitations to this work. It remains uncertain whether our prepared hydrogel is effective for the management of high-voltage, high-current electricity-induced full-thickness burn injuries with large areas of burn surface and complex limb trauma. Epidemiological data indicate that high-voltage, high-current injuries bring more catastrophic consequences, representing a critical clinical imperative that warrants urgent intervention.

Based on its favorable thermosensitivity, this study focuses on injectable formulation of the CS/AST/IBU hydrogel for treatment of high-voltage, low-current electrical burns. Regarding the prepared hydrogel co-loaded with AST/IBU for reactive oxygen species (ROS) scavenging and NF-κB pathway inflammation inhibition, respectively, it may have a broad of biomedical applications when it is developed as other alternative formulations (e.g., topical gels, dressings). Particularly, in clinical scenarios where injection is contraindicated, the hydrogel can be straightforwardly used as topical gels, ointments, or adhesive patches for non-invasive application [42]. It can also be processed into thermosensitive adhesive patches or semi-solid ointments that undergo in situ gelation upon contact with skin, forming a conformal, protective barrier that maintains a moist wound environment while sustained release of AST and IBU [72]. Such formulations would preserve the hydrogel’s ability to modulate oxidative stress and inflammation, making them ideal for outpatient wound care. The intrinsic antimicrobial activity of chitosan (CS), coupled with the anti-inflammatory IBU and antioxidant AST, may offer this hydrogel as a potential candidate for treating chronic, hard-healing wounds, such as diabetic foot ulcers and venous leg ulcers, in which persistent oxidative stress and dysregulated inflammation severely impede tissue regeneration [73].

Furthermore, a longer observation period would be more suitable for assessing full burn remodeling. We recognize that comprehensive evaluation of scar quality—including collagen architecture and dermal appendage regeneration—requires extended observation periods, which will be addressed in future studies using histological techniques such as picrosirius red staining and scar elevation index measurements. Additionally, while the inclusion of ibuprofen suggests potential analgesic benefits, pain behavior was not directly assessed in this study; future investigations should incorporate behavioral assays (e.g., mechanical withdrawal threshold) to validate analgesic efficacy. Finally, to enhance translational relevance, subsequent studies should include clinically used dressings (e.g., silver sulfadiazine) as comparators.

## 5. Conclusions

In this study, we reported the design, validation, and mechanism analysis of a multifunctional thermoresponsive polysaccharide hydrogel (CS/AST/IBU) engineered for the advanced treatment of skin electrical burn injuries. This injectable hydrogel presents intrinsic anti-inflammatory and antioxidant properties that synergistically promote vascular endothelial angiogenesis, leading to markedly enhanced wound closure. Crucially, in a rat model of high-voltage, low-current skin electrical burn injury, the CS/AST/IBU hydrogel significantly accelerated wound healing. Mechanistic investigations revealed that this pro-regenerative effect was orchestrated via suppression of the pro-inflammatory ROS–NF-κB–inflammatory axis. Taken together, the results of this work establish the proposed hydrogel as a novel therapeutic platform that may address the complex challenges associated with the treatment of skin electrical burn injuries.

## Data Availability

The raw data supporting the conclusions of this article will be made available by the authors on request.

## Supplementary Materials

The following supporting information can be downloaded at: https://www.mdpi.com/article/10.3390/pharmaceutics18030323/s1, Figure S1: Histopathological evaluation of SD rats’ skin at 24 h post-injury (H&E staining). Figure S2: Hierarchical clustering heatmap of the DEPs in control and CS/AST/IBU treatment groups. Figure S3: Hierarchical clustering heatmap of the DEPs in control and CS treatment groups. Figure S4: Volcano plot of the DEPs. Figure S5: GO enrichment analysis of the DEPs. Figure S6: Western blot image with molecular weight marker. Table S1: Antibodies used in this study. Table S2: Drug release kinetics fitting parameters for IBU and AST from CS/AST/IBU hydrogel at 37 °C. Table S3: Quantitative analysis of EPR spectra for free radical scavenging activity.

## Abbreviations

ABTS	2,2′-azinobis-(3-ethylbenzthiazoline-6-sulfonate)
AST	astaxanthin
BCA	bicinchoninic acid
BSA	bovine serum albumin
CAT	catalase
CCK-8	Cell Counting Kit-8
CS	chitosan
DCM	dichloromethane
DPPH	1,1-diphenyl-2-picryl-hydrazyl radical
ECL	chemiluminescence
ESR	electron spin resonance
EVG	Elastica van Gieson
FTIR	Fourier transform infrared
GO	Gene Ontology
GSH	glutathione
H&E	hematoxylin and eosin
HaCaT cells	human immortalized epidermal cells
HPCD	hydroxypropyl-β-cyclodextrin
HPMC	hydroxypropyl methylcellulose
HRP	horseradish peroxidase
IBU	ibuprofen
IF	immunofluorescence
IHC	immunohistochemistry
KEGG	Kyoto Encyclopedia of Genes and Genomes
LCST	lower critical solution temperature
MDA	malondialdehyde
NF-κB	nuclear factor kappa-B
PCA	principal component analysis
PVDF	polyvinylidene difluoride
RIPA	radioimmunoprecipitation assay
ROS	reactive oxygen species
SDS-PAGE	SDS-polyacrylamide gel electrophoresis
SEM	scanning electron microscopy
SOD	superoxide dismutase
β-CD	β-cyclodextrin
