# Supplementary material for: An Injectable Thermosensitive Chitosan/Astaxanthin/Ibuprofen Hydrogel Mitigates High-Voltage, Low-Current Electrical Burn Injury Through Inhibition of ROS–NF-κB Signaling-Mediated Inflammation
Source: Pharmaceutics. 2026 Mar 3;18(3):323. doi: 10.3390/pharmaceutics18030323 (PMC13029109; doi:10.3390/pharmaceutics18030323)
Supplement: Supplementary file 1 [file pharmaceutics-18-00323-s001.zip › pharmaceutics-4166982-supplementary.pdf]

Supplementary data

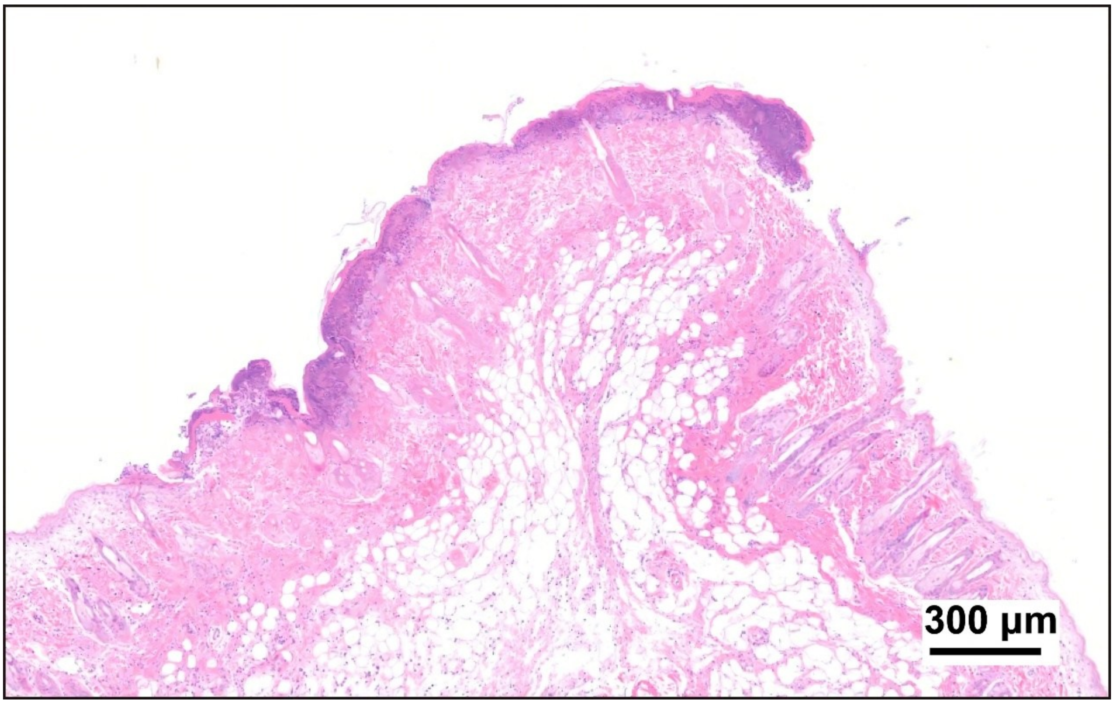

Figure S1. Histopathological evaluation of SD rats' skin 24 h post-injury (H&E staining).

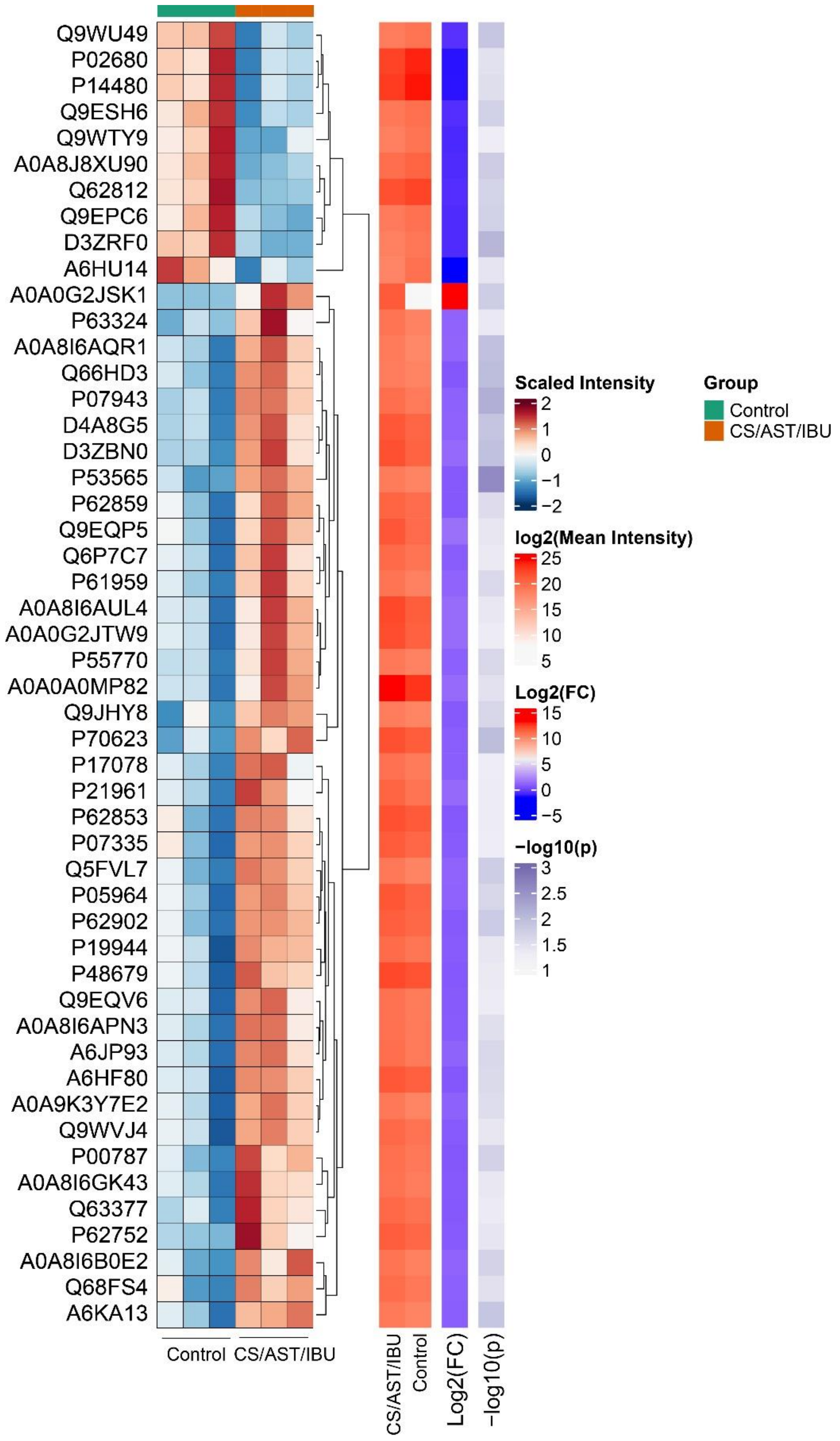

Figure S2. Hierarchical clustering heatmap of the DEPs in control and CS/AST/IBU treatment groups.

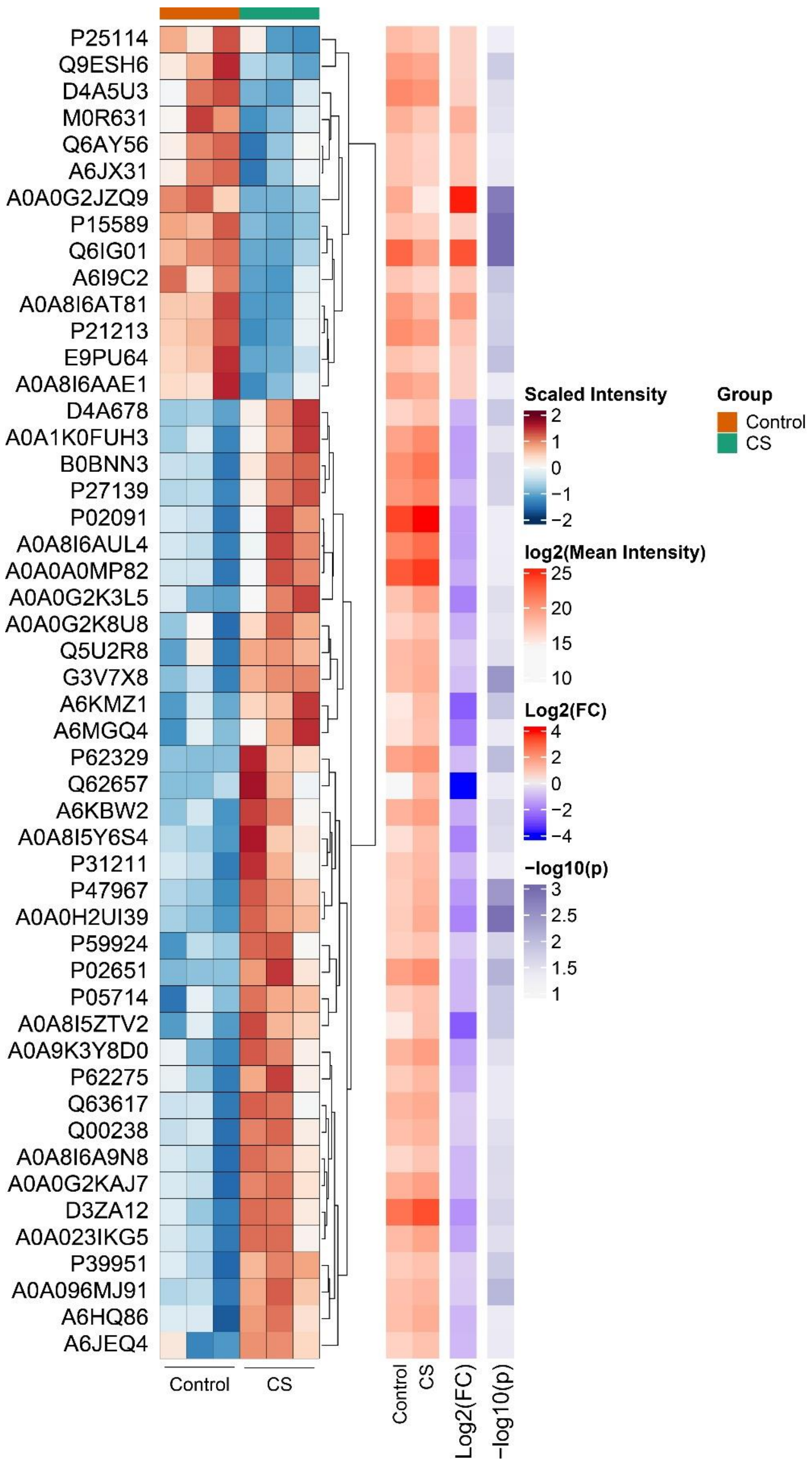

Figure S3. Hierarchical clustering heatmap of the DEPs in control and CS treatment groups.

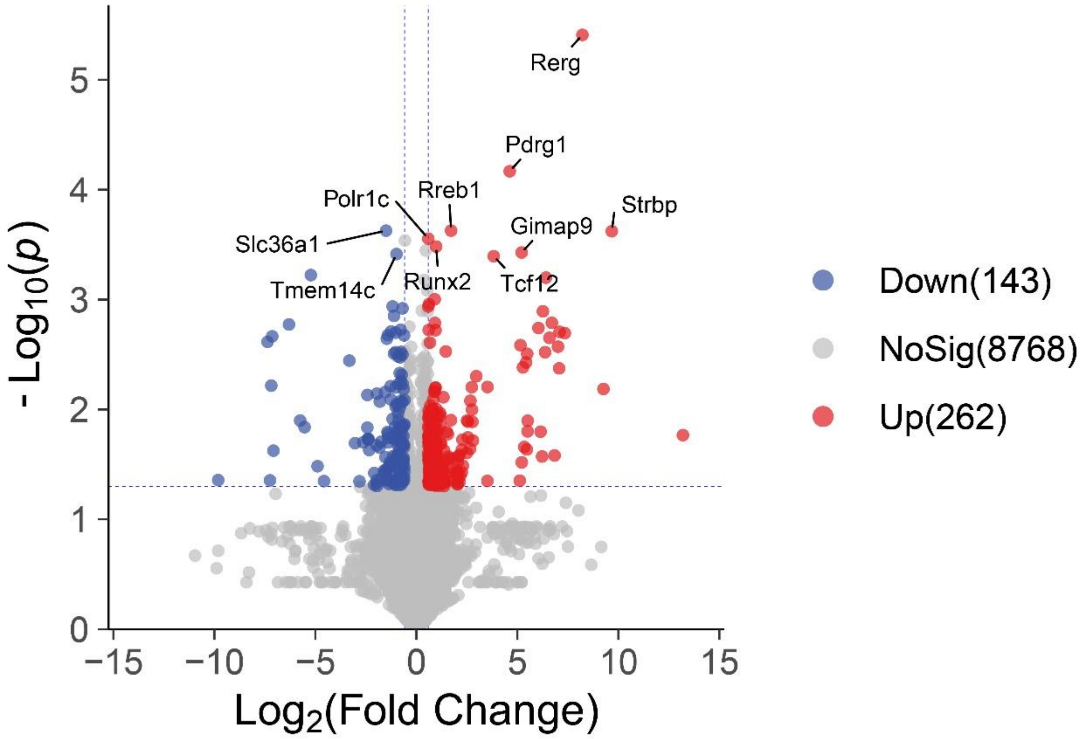

Figure S4. Volcano plot of the DEPs

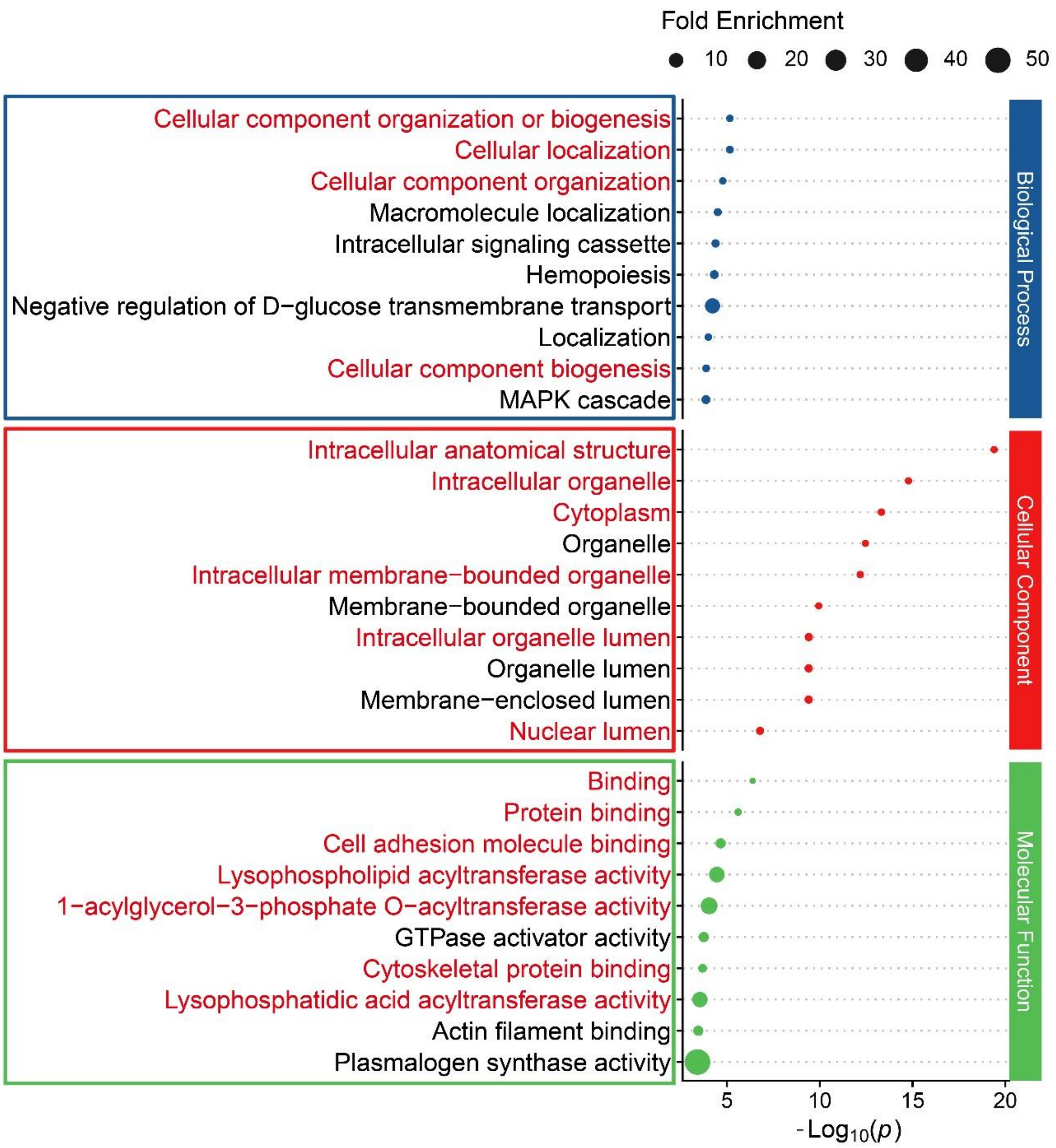

Figure S5. GO enrichment analysis of the DEPs.

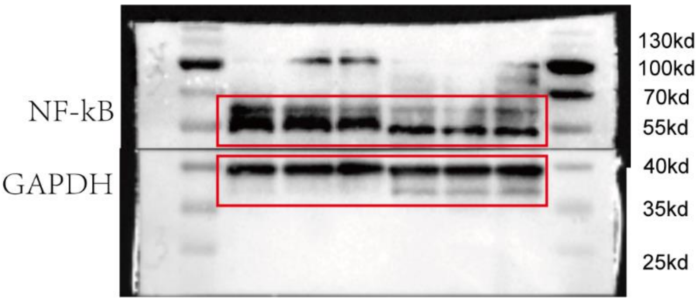

Figure S6. The protein bands along with the molecular weight (band size) marker.

Table S1. Antibodies used in this study

Antigen	Catalogue number	Supplier	Dilution ratio	Host species	Application
CD11B	GB15058	Servicebio	1:500	Rab	Immunohistochemistry
CD163	GB15340	Servicebio	1:500	Rab	Immunohistochemistry
IL-1b	GB11113	Servicebio	1:500	Rab	Immunohistochemistry
TNF-a	GB115701	Servicebio	1:500	Rab	Immunohistochemistry
CD31	GB120008	Servicebio	1:500	Rab	Immunofluorescence
α-sma	GB111364	Servicebio	1:500	Rab	Immunofluorescence
NF κB P65	AB2020	Beyotime	1:1000	Rab	Western blot
	GB12997	Servicebio	1:500	Mus	Immunohistochemistry
GAPDH	AF1186	Beyotime	1:1000	Rab	Western blot
DAPI	G1012	Servicebio			Immunofluorescence
Goat Anti-Mouse IgG (HRP)	A0216	Beyotime	1:1000	Goat	Western blot
Goat Anti-Rabbit IgG (HRP)	Ab205718	Abcam	1:2000	Goat	Western blot
Alexa Fluor™ 568, Donkey anti-Mouse IgG	A10037	Invitrogen	1:200	Donkey	Immunohistochemistry
Alexa Fluor™ 488, Donkey anti-Rabbit IgG	A21206	Invitrogen	1:200	Donkey	Immunohistochemistry
Alexa Fluor™ 568, Donkey anti-Rat IgG	A78946	Invitrogen	1:200	Donkey	Immunohistochemistry
Alexa Fluor™ 488, Goat anti-Rabbit IgG	GB25303	Servicebio	1:200	Goat	Immunofluorescence
Goat Anti-Mouse IgG (HRP)	GB23301	Servicebio	1:200	Goat	Immunofluorescence

Table S2. Drug release kinetics fitting parameters for IBU and AST from CS/AST/IBU hydrogel at 37°C.

Drug	Model	Equation	k (unit)	n	R^2
IBU	Zero-order	Q = kt	0.678 %·h^{-1}	–	0.938
IBU	First-order	ln(1-Q/100) = –kt	0.0099 h^{-1}	–	0.887
IBU	Higuchi	$Q = k \cdot t^{1/2}$	7.01 %·$h^{-1/2}$	–	0.955
IBU	Korsmeyer–Peppas	$Q = k \cdot t^{n}$	42.3 %·h^{-n}	0.68	0.994
AST	Zero-order	Q = kt	0.58 %·h^{-1}	–	0.940
AST	First-order	ln(1-Q/100) = –kt	0.0085 h^{-1}	–	0.902
AST	Higuchi	$Q = k \cdot t^{1/2}$	5.89 %·$h^{-1/2}$	–	0.953
AST	Korsmeyer–Peppas	$Q = k \cdot t^{n}$	32.8 %·h^{-n}	0.65	0.996

Note: The release exponent n from the Korsmeyer–Peppas model is used to determine the release mechanism. For cylindrical geometry, $n<0.45$ indicates Fickian diffusion, $0.45<n<0.89$ indicates anomalous (non-Fickian) transport, and $n>0.89$ indicates Case-II transport. The n values for IBU (0.48) and AST (0.52) fall within the anomalous transport range, indicating that drug release is governed by both diffusion and polymer chain relaxation, which is consistent with the thermosensitive gelation behavior of the hydrogel.

Table S3. Quantitative Analysis of EPR Spectra for Free Radical Scavenging Activity

**Parameter**	**Superoxide Radical ($O_2^{\bullet-}$)**	**Hydroxyl Radical (•OH)**
**Control Group**		
Maximum Positive Intensity	72,771.58	93,510.94
Minimum Negative Intensity	-65,390.31	-92,137.67
Peak-to-Peak Amplitude	138,161.89	185,648.61
**Experimental Group (CS/AST/IBU H)**		
Maximum Positive Intensity	16,028.63	20,777.04
Minimum Negative Intensity	-14,342.81	-19,474.23
Peak-to-Peak Amplitude	30,371.44	40,251.27
Reduction Calculation		
Percentage of Control Signal	22.0%	21.7%
**Radical Scavenging Efficiency**	**78.0%**	**78.3%**
